# SIRT6 in Aging, Metabolism, Inflammation and Cardiovascular Diseases

**DOI:** 10.14336/AD.2022.0413

**Published:** 2022-12-01

**Authors:** Zhenyang Guo, Peng Li, Junbo Ge, Hua Li

**Affiliations:** ^1^Department of Cardiology, Zhongshan Hospital, Shanghai Institute of Cardiovascular Diseases, Fudan University, Shanghai, China.; ^2^Institutes of Biomedical Sciences, Fudan University, Shanghai, China

**Keywords:** SIRT6, molecular network, ageing, metabolism, inflammation, cardiovascular diseases

## Abstract

As an important NAD^+^-dependent enzyme, SIRT6 has received significant attention since its discovery. In view of observations that SIRT6-deficient animals exhibit genomic instability and metabolic disorders and undergo early death, SIRT6 has long been considered a protein of longevity. Recently, growing evidence has demonstrated that SIRT6 functions as a deacetylase, mono-ADP-ribosyltransferase and long fatty deacylase and participates in a variety of cellular signaling pathways from DNA damage repair in the early stage to disease progression. In this review, we elaborate on the specific substrates and molecular mechanisms of SIRT6 in various physiological and pathological processes in detail, emphasizing its links to aging (genomic damage, telomere integrity, DNA repair), metabolism (glycolysis, gluconeogenesis, insulin secretion and lipid synthesis, lipolysis, thermogenesis), inflammation and cardiovascular diseases (atherosclerosis, cardiac hypertrophy, heart failure, ischemia-reperfusion injury). In addition, the most recent advances regarding SIRT6 modulators (agonists and inhibitors) as potential therapeutic agents for SIRT6-mediated diseases are reviewed.

Sirtuins, comprising a group of evolutionarily conserved nicotinamide adenine dinucleotide (NAD^+^)-dependent proteins, beneficially regulate lifespan and cellular senescence [1]. Interestingly, in contrast to class I, II and IV HDACs, for which Zn^2+^ is a catalytic cofactor, the dependence of sirtuins on NAD^+^ distinguishes them from other classes of HDACs; sirtuins are class III HDACs [2]. In mammalian cells, seven different sirtuin proteins have been identified (SIRT1-7) [3]. They share a conserved ~270 residue catalytic core domain composed of an NAD^+^-binding Rossmann subdomain and a Zn^2+^-binding module, with variable N- and C-terminal extensions (NTEs and CTEs, respectively) of different lengths and sequences, which account for their specific localization, unique substrates and multiple physiological functions [4]. SIRT1 and SIRT2 are present in both the nucleus and cytoplasm; SIRT3, SIRT4 and SIRT5 are exclusively found in mitochondria, and SIRT6 and SIRT7 are thought to be located in the nucleus [3]. Notably, in response to stress, SIRT6 localizes to cytoplasmic stress granules [5, 6], suggesting that SIRT6 is not exclusively a nuclear protein. Sirtuins are involved in a broad range of physiological processes, including genome stability, energy metabolism, aging, tumorigenesis, and cardiovascular biology, via their regulation of key protein activities [3, 7].

Among sirtuin family members, sirtuin 6 (SIRT6) is of particular interest and has gained more attention due to its distinctive enzymatic activities; for example, SIRT6 catalyzes deacetylation and mono-ADP-ribosylation and exhibits long-chain fatty acid (FA) deacylase activity [6]. These enzymatic activities indicate that SIRT6 is closely related to cellular biological processes, such as DNA repair, genome stability, inflammation and metabolic homeostasis [6]. Studies have revealed that dysregulation of SIRT6 activity leads to the onset and development of many diseases, including but not limited to metabolic diseases, cardiovascular diseases (CVDs), cancers and neurodegenerative diseases [8]. Therefore, a thorough and detailed understanding of the roles and regulatory mechanisms of SIRT6 may pave a new way to novel therapeutic interventions for these diseases.

In this review, we first focus on the distinctive molecular structure and biological functions of SIRT6 and then illustrate recent advances in understanding the involvement of SIRT6 in cell and molecule signaling pathways related to senescence, dysregulated metabolism, inflammation and oxidative stress. In particular, we highlight the critical roles of SIRT6 in CVDs and their related risk factors and discuss recent developments in SIRT6 modulators, their pharmacological profiles with respect to their potential use as therapeutics.

## 1. The structure and enzymatic activities of SIRT6

### 1.1 Structural features of SIRT6

**Figure 1.**
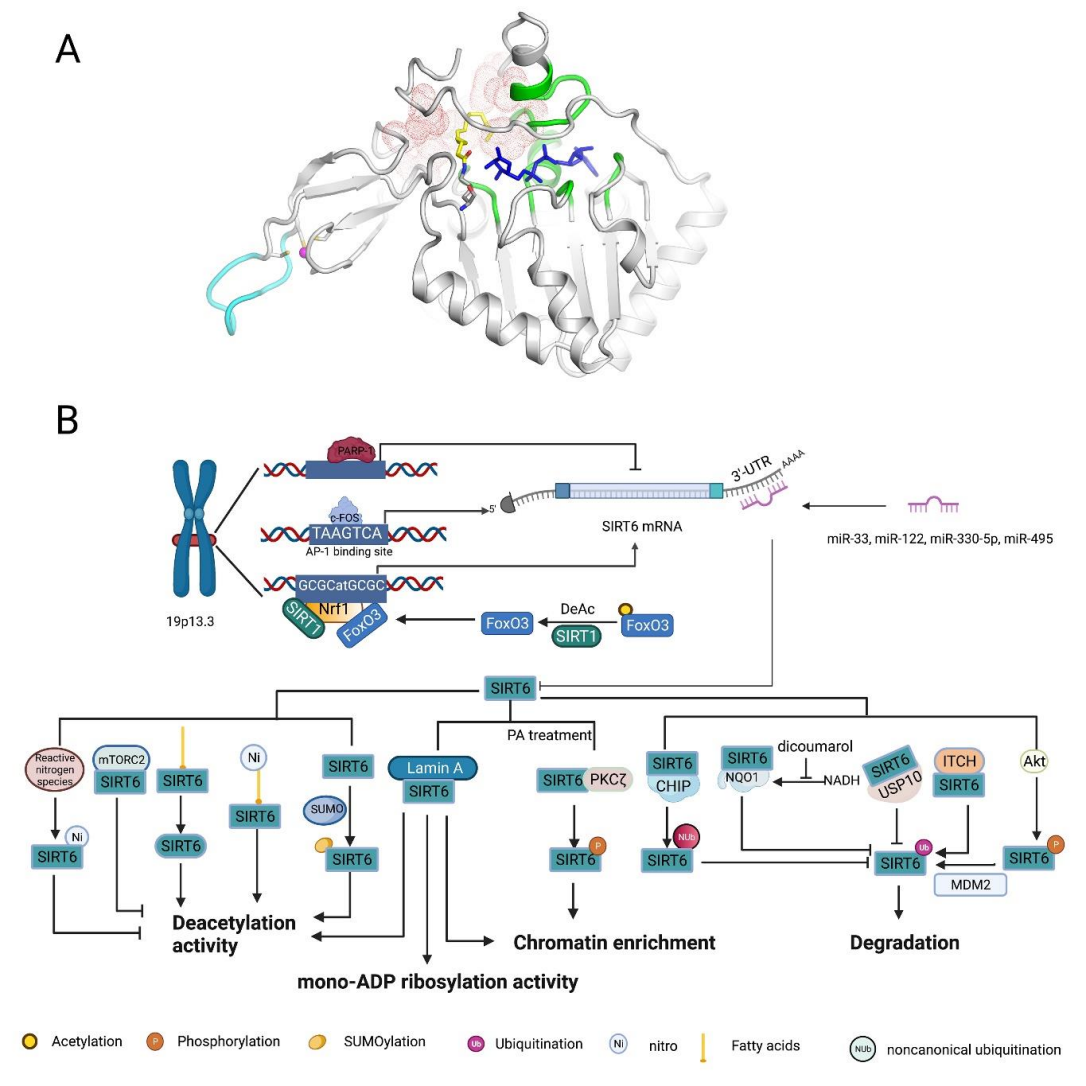
The structural features and molecular regulation of SIRT6. (A) The structural features of SIRT6: Structure of human SIRT6 in complex with H3K9-Myr (yellow) and ADP-ribose (blue) bound (PDB ID: 3ZG6). The zinc ion (pink)-binding structure is shown in the bottom left, and the unique flexible loop in the zinc-binding module is shown in azure. The stable structure of NAD+ binding with SIRT6 is colored green, and the long and wide hydrophobic-channel pocket is shown as red dots. (B) At the transcriptional level, p53 and pharmacological inhibition of PARP1 both upregulate SIRT6 expression. Similarly, c-FOS binds to the AP-1-binding site (TAAGTCA) in the SIRT6 promoter to directly promote SIRT6 expression. Under nutrient stress, SIRT1 interacts with and deacetylates FOXO3a, which is favorable for the formation of the SIRT1-FOXO3a-NRF1 (SFN) complex on the promoter of SIRT6, which upregulates SIRT6 expression. In addition, endogenous microRNAs (miRNAs), such as miR-33, miR-122, miR-330-5p and miR-495, silence translation by binding to the 3’-untranslated region (UTR) of SIRT6 mRNA. At the protein level, fatty acids (FAs) and nitrated FAs can activate SIRT6 deacetylase activity. The binding of electrophilic nitro-FAs and SIRT6 induces efficient activation (40-fold at 20 μM). In contrast, reactive nitrogen-induced nitration of tyrosine 257 (Y257) in SIRT6 causes loss of SIRT6 activity. Interaction between mTORC2 and SIRT6 suppresses SIRT6 deacetylase activity in adipose tissue. Lamin A is an endogenous activator of SIRT6, promoting SIRT6 recruitment to chromatin and activating both its deacetylase and mono-ADP ribosyltransferase activity. In addition, SUMOylation of SIRT6 specifically regulates SIRT6 deacetylation on H3K56 *in vivo*, and four lysine residues of SIRT6, K296, K300, K316 and K332, are thought to be SUMOylated. Under PA treatment, PKCζ binds to SIRT6 and phosphorylates SIRT6 at threonine 294 (T294) to promote SIRT6 recruitment to chromatin. Regarding stability, Akt-mediated phosphorylation of SIRT6 at serine 338 (S338) makes SIRT6 favorable for the ubiquitination via MDM2, promoting SIRT6 degradation. In contrast, SIRT6 interaction with USP10, CHIP and NQO1 blocks ubiquitin-mediated degradation of SIRT6. Among these factors, CHIP induces noncanonical ubiquitination of SIRT6 at K170, preventing canonical ubiquitination by other ubiquitin ligases. In addition, the NQO1 cofactor NADH promotes the binding of NQO1 to SIRT6, whereas DIC compromised the interaction between NQO1 and SIRT6. Ni, nitration; SUMOylation, SUMO-induced modification; Nub, noncanonical ubiquitination; Ub, ubiquitination.

Characterizing the molecular functions of SIRT6 has been challenging, mainly because this protein can participate in three different enzymatic activities with diverse substrates [9]. Fortunately, detailed structural studies have provided in-depth insights into the characteristics of SIRT6, such as its subcellular localization, catalytic activity, and substrate preference. The N-terminus of SIRT6 is essential for chromatin association and intrinsic histone 3 lysine 9 (H3K9) and H3K56 deacetylation activity, whereas the C-terminus is required for the nuclear localization and recognition of nucleosomal DNA [10, 11]. In the catalytic core, Rossmann fold subdomain of SIRT6 contains a stable helical structure for NAD^+^ binding; however, most sirtuins are characterized by a highly flexible NAD^+^-binding loop [12]. This distinctive structure of SIRT6 favors high affinity binding with NAD^+^ even in the absence of acetylated substrates, which may be the reason that SIRT6 can efficiently promote mono-ADP-ribosyltransferase reactions [12]. As mentioned above, Zn^2+^ plays a purely structural role and is coordinated by SIRT6 Cys141, Cys144, Cys166, and Cys177 in the small and splayed zinc-binding module, in which ten residues (167-176) form a flexible loop that is unique to SIRT6 [13]. Mutation of Cys144 was experimentally shown to decrease SIRT6 deacetylase activity and significantly promote its glycolytic capacity and lead to the accumulation of extracellular lactate but did not decrease glucose transporter-1 (GLUT-1) expression, suggesting that Cys144 of SIRT6 is potentially a critical site in SIRT6 regulation of glucose metabolism [14]. Additionally, several other mutations of residues that impair SIRT6 functions have been identified: The H133Y mutation results in abrogation of SIRT6 catalytic activity and impaired SIRT6 binding with chromatin; the S56Y mutant lacks both deacetylase activity and mono-ADP-ribosyltransferase activity; R65A leads to abrogated deacetylase activity, but mono-ADP-ribosyltransferase activity is retained, while the G60A mutation has the opposite effect. In addition, a long active site is found between the Rossmann and Zn^2+^ subdomains of SIRT6; it is the widest hydrophobic channel pocket in sirtuins [12], which may explain the reason that SIRT6 prefers long-chain fatty acylation to acetylation, at least *in vitro* [15-17] (Fig. 1A).

### 1.2 Enzymatic activities of SIRT6

#### 1.2.1 Deacetylase activity

A breakthrough in understanding the molecular functions of SIRT6 came with the discovery of its deacetylase activity against H3K9 and H3K56 [18, 19], with this discovery leading to a series of studies on the roles of SIRT6 in regulating gene expression and telomeric chromatin stability and its effects on the dynamic association of DNA repair factors with chromatin [20]. Later studies revealed that SIRT6 deacetylates H3K18, which is required for the maintenance of pericentric heterochromatin and repression of pericentric transcripts, thereby protecting against mitotic errors and cellular senescence [21]. SIRT6 also actively deacetylates H3K27, but the specific molecular mechanisms involved in this modification need to be further explored [22]. While the histone deacetylase activity of SIRT6 has been well characterized, SIRT6 has been shown to engage in deacetylase activity at a rate ~3 orders of magnitude slower than that of other sirtuins *in vitro* [15]. Furthermore, Feldman *et al*. showed that SIRT6 deacetylation activity was positively upregulated by long-chain FAs, providing an explanation for the discrepancy between its poor deacetylase activity *in vitro* and the clear observation that SIRT6 is a histone deacetylase *in vivo* [23]. Another explanation suggests that SIRT6 histone deacetylase activity depends on packaged nucleosomes, not free histone proteins; in other words, the SIRT6 deacetylase structure can be converted upon binding to nucleosomes [24]. Recently, SIRT6 has been found to directly detach acetyl groups from some nonhistone proteins, a regulatory function important to certain cellular biological processes. For example, SIRT6 directly deacetylates general control nonrepressed protein 5 (GCN5) and pyruvate kinase M2 (PKM2), thereby regulating their enzymatic activities [25, 26]. SIRT6 also increases cellular NADPH levels by directly deacetylating nicotinamide phosphoribosyltransferase (NAMPT), conferring cellular resistance to oxidative stress damage [27]. The active spliced form of X-box-binding protein 1 (XBP1) is deacetylated by SIRT6, which leads to XBP1 degradation and subsequent prevention of endoplasmic reticulum (ER) stress-induced hepatic steatosis [28]. Recently, SIRT6 has also been shown to deacetylate SMAD2 and SMAD3, attenuating the development of liver fibrosis [29, 30].

#### 1.2.2 Mono-ADP-ribosyltransferase activity

Although the deacetylase activity of SIRT6 has been well established, its first discovered enzymatic activity is the NAD^+^-dependent mono-ADP-ribosyltransferase activity [31]. In an *in vitro* experiment, SIRT6 was shown to transfer radiologically labeled [^32^P] NAD^+^ onto itself, which indicated that SIRT6 underwent autoregulation via mono-ADP-ribosylation, however, the physiological significance of this auto-ADP ribosylation remains unknown [31]. Recent studies identified several mono-ADP-ribosylation substrates of SIRT6, including KAP1, PARP1, KDM2A and BAF170, which are mainly involved in the aging process, genome stability maintenance and oxidative stress [32-35]. Moreover, findings by Van Meter *et al*. suggested that the mono-ADP-ribosyltransferase activity of SIRT6 was involved in the regulation of the apoptosis of cancer cells by activating the p53 and p73 signaling cascades [36]. Notably, previous studies reported that the mono-ADP-ribosylation activity of SIRT6 was negligible *in vitro*, suggesting that this activity depended on specific *in vivo* substrates or conditions [9].

#### 1.2.3 Long-chain FA deacylase activity

The weak deacetylation activity of SIRT6 and its preference for long-chain fatty acylation *in vitro* led to the speculation that SIRT6 was engaged in other enzymatic activities in addition to its established deacetylation and mono-ADP-ribosylation functions. Indeed, SIRT6 can efficiently catalyze long-chain FA deacylation, and the most notable effect of this function is associated with protein secretion [37]. One representative protein, TNF-α, is secreted and regulated by SIRT6 demyristoylation [15]. Furthermore, SIRT6 has also been shown to change the subcellular localization of R-Ras2 by regulating its depalmitoylation, inhibiting activation of the PI3K/Akt signaling pathway [38]. An *in vitro* experiment also showed that the FA deacylation of myristoylated, palmitoylated and octanoylated peptides in H3K9 and that of a myristoyl group on H2BK12 was an efficient function of SIRT6 [15]. Another biological study confirmed that SIRT6 effectively removed fatty acyl groups from histones H3K9, H3K18, and H3K27 and from histones H3K14, H3K36, H3K56 and H3K79 at a relatively low rate [22]. This discovered enzymatic function of SIRT6 may be involved in regulating chromatin accessibility and gene transcription, but the exact potential physiological functions remain to be further elucidated.

## 2. The molecular network that regulates SIRT6

The abundance of SIRT6 is subjected to intricate and precise regulation *in vivo* (Fig. 1B). At the transcriptional level, pharmacological inhibition of poly-ADP-ribose polymerase 1 (PARP1) significantly activates the transcription of *SIRT6* [39]. Additionally, c-FOS and the SIRT1-FOXO3a-NRF1 (SFN) complex bind the SIRT6 promoter to upregulate *SIRT6* expression [40, 41]; the tumor suppressor p53 has also been shown to activate the expression of *SIRT6* [42]. In contrast, both high glucose levels *in vitro* and maternal diabetes *in vivo* significantly reduced the SIRT6 level in the embryo or neural stem cells [43]. Interestingly, a separate study demonstrated that high glucose exposure was associated with increased demethylation of the SIRT6 promoter and increased SIRT6 mRNA transcription [44]. Translation or posttranslational modifications might be feasible explanations for the discrepancy observed in the regulatory mechanisms of SIRT6. At the translational level, endogenous microRNAs (miRNAs), particularly miR-33a/b, miR-122, miR-330-5p and miR-495, can regulate SIRT6 translation by binding the 3’-untranslated region (UTR) of SIRT6 [45-48]. At the protein level, the stability of SIRT6 is most notably regulated through the proteasome-dependent degradation pathway. Previous studies showed that Akt-induced phosphorylation and E3 ubiquitin ligase ITCH-induced ubiquitination of SIRT6 both accelerated its degradation [49, 50]. In contrast, the ubiquitin ligase CHIP and ubiquitin-specific peptidase USP10 acted as protective regulators to maintain SIRT6 stability [51, 52]. In addition, NAD(P)H: quinone oxidoreductase 1 (NQO1) has been shown to physically interact with SIRT6 to prevent its degradation [53].

SIRT6 deacetylase activity can be activated by binding of several specific long-chain fatty FAs (myristic, oleic, and linoleic acids) *in vitro*, suggesting that regulatory pathways are involved in SIRT6 enzymatic activities, at least its deacetylase activity [23]. Mechanistically, FAs bind to the hydrophobic pocket of SIRT6 to induce a conformational change in SIRT6 that increases its affinity for acetylated substrates. Recent research has revealed that more efficient nitro-fatty acids (NO2-FA) binding stimulates SIRT6 deacetylase activity in a cellular environment [13]. In contrast, peroxynitrite and other nitric oxide-derived oxidants have been shown to modify tyrosine and cysteine residues in SIRT6, resulting in impaired SIRT6 deacetylase activity [54, 55]. Similarly, mTORC2 interacts with SIRT6 to suppress its deacetylation activity against FoxO1 [56]. In addition, small ubiquitin-like modifier (SUMO) has been shown to modify SIRT6 and specifically upregulate its deacetylation of H3K56 but not H3K9 [57]. Atypical protein kinase C ζ (PKCζ) phosphorylates SIRT6 to promote its enrichment on the promoters of β-oxidation-related genes [58]. Both deacetylation of SIRT6 by SIRT1 and the binding of SIRT6 to Lamin A promote the retention of SIRT6 on DNA breaks to promote DNA repair [59, 60]. In addition, Lamin A binds nuclear factor erythroid 2-related factor 2 (Nrf2) and SIRT6 and stimulates both the deacetylation and mono-ADP ribosylation activities of SIRT6, promoting the proper expression of NRF2 target genes [61].

In summary, stress conditions, cellular metabolites and other stimuli have been proven to finely tune the abundance and enzymatic activity of SIRT6 in multiple ways. A previous study showed that SIRT6 was phosphorylated at conserved residues (T294, 303, S330, and S338) within the C-terminus, suggesting that the C-terminus modification patterns may be crucial to SIRT6 functions [62]. As mentioned above, in the C-terminus, both Akt- and PKCζ-induced phosphorylation and SUMO-induced SUMOylation affected SIRT6 abundance and functions. However, the effects of other modifications at C terminal sites remain to be further studied. Considering the diverse roles of SIRT6 in numerous molecular signaling pathways, some of the aforementioned regulatory factors may be feasible intervention targets for regulating SIRT6-mediated signaling.

## 3. The biological functions of SIRT6

Among the diverse functions of SIRT6, its most remarkable role is an intricate regulator of cell senescence and lifespan. Owing to genome instability and dysregulated metabolism, *Sirt6*-deficient mice exhibit aging-associated degenerative phenotypes, including severe metabolic disorders, subcutaneous fat loss, and lordokyphosis, finally dying at about 4 weeks old; in contrast, transgenic mice overexpressing *Sirt6* show a longer lifespan than their wild-type counterparts [63, 64]. Furthermore, the contributions of SIR6 to genome stability, DNA repair and telomere function highlight its important therapeutic potential for aging and aging-related diseases [9]. In addition, oxidative stress, inflammation, and metabolism, in which SIRT6 is involved, are crucial for the occurrence and development of CVDs [65]. Moreover, SIRT6 plays essential roles in protecting vessels and heart from endothelial dysfunction [66], atherosclerosis [67], pathological cardiac hypertrophy [68], myocardial fibrosis [69], heart failure [70] and ischemia/reperfusion (I/R) injury [71]. Notably, in some cases, SIRT6 appeared to be a pernicious regulator. For example, overexpression of SIRT6 significantly promoted angiogenesis and hemorrhage in carotid plaques, resulting in plaque instability [72]. In addition, in a mouse model, *Sirt6* silencing reduced neutrophil infiltration, myocardial infarct size and reactive oxygen species (ROS) generation within infarcted heart tissues in the early phases of I/R [73]. Given the important and diverse enzymatic effects of SIRT6 on cellular physiological functions, it is of significance to discuss the mechanisms by which these pleiotropic SIRT6 functions extensively affect biological functions and the determination of whether SIRT6 is a feasible therapeutic target for aging-related diseases. Detailed information on SIRT6 substrates and functions is shown in Table 1.

### 3.1 SIRT6 inhibits senescence by maintaining genome stability

Although as an inevitable part of human life, cellular senescence remains a significant risk factor for age-related diseases [74]. Notably, numerous studies have shown that older mammals are more likely to suffer from DNA damage than younger mammals. However, in progeroid syndromes, defects in cellular responses to DNA damage can accelerate the aging process; that is, to some extent, DNA damage is probably a cause, not a consequence of aging, highlighting the importance of DNA damage in the aging process [75]. SIRT6 attenuates cell aging by maintaining genome stability. For example, SIRT6-mediated stabilization of repressive hetero-chromatin in subtelomeric regions results in the silencing of telomere-proximal genes, which is important to genome stability maintenance [76]. In addition, both the deacetylation and mono-ADP ribosylation activities of SIRT6 have been shown to promote DNA repair through multiple pathways [8]. Importantly, SIRT6, which maintains genome integrity, for example, by preventing DNA damage and promoting DNA repair, has been implicated in CVDs [77]. Previous works have demonstrated that SIRT6 protects endothelial cells (ECs) and vascular smooth muscle cells (VSMCs) from telomere and genomic DNA damage, thus preventing the onset of senescence [78, 79]. Therefore, we mainly elaborate on the specific mechanisms of SIRT6 in genomic damage to highlight its protective roles of SIRT6 during aging.

**Table 1:** The substrates and functions of SIRT6.

SIRT6 function	Substrates		Molecular mechanism	Physiological functions
Deacetylase	Histones	H3K9	Inhibits NF-kB target gene expression	Prevents premature aging [160]
			Inhibits 53BP1 binding to telomeres	Prevents telomere damage and delays VSMC senescence [78]
			Facilitates loading of CHD4 onto DNA damage sites	Promotes chromatin relaxation and subsequent DNA repair [108]
			Inhibits Nkx3.2 expression and thereby promotes GATA5 expression	Prevents hypertension and associated cardiorenal injury [66]
			Inhibits HIF1α and associated glycolytic gene expression	Improves mitochondrial respiration and inhibits glycolysis against metabolic diseases [111]
			Inhibits Notch1 and Notch4 expression	Protects podocytes from injury and attenuates proteinuria [245]
			Inhibits expression of the proatherogenic gene TNFSF4	Maintains endothelial function and protects against atherosclerosis [246]
			Inhibits IGF signaling-related gene expression	Restricts the development of cardiac hypertrophy [68]
			Inhibits c-Jun-dependent proinflammatory gene expression	Attenuates chronic liver inflammation and fibrosis [247]
			Inhibits ERK1/2 expression	Attenuates cisplatin-induced kidney injury [248]
			Facilitates binding of WRN with telomeres	Protects against telomere dysfunction and premature ageing disorders [249]
			Inhibits NKG2D ligand expression in ECs	Stabilizes atherosclerotic plaques and restricts atherosclerosis [179]
			Inhibits *Txnip* expression in β cells	Maintains pancreatic β-cell function and viability [124]
		H3K56	Inhibits β-catenin-dependent pro-fibrotic gene expression	Protects against kidney fibrosis following kidney ischemia-reperfusion injury [250]
			Facilitates recruitment of the chromatin remodeler SNF2H to DSBs	Promotes DSB repair and genomic stability [236]
			Facilitates recruitment of DNA repair proteins	Promotes the DDR process [106]
			Inhibits catalase expression	Promotes neovascular injury by increasing ROS [72]
			Promotes assembly of the Nrf2-RNAPII transcription complex at the HO-1 promoter, upregulating HO-1 expression	Facilitates ROS clearance to counteract oxidative stress injury [154]
		H3K18	Reduces levels of activated RNA Pol II and H3K36me3	Protects against mitotic errors and cellular senescence [21]
	Non- histones	SMAD2 (K54)	Reduces the transcriptional activity of SMAD2	Protects against liver fibrosis [29]
		SMAD3 (K333/378)	Reduces the transcriptional activity of SMAD3	Protects against liver fibrosis [30]
		P53 (K381/382)	Inhibits P53 transcriptional activity	Ameliorates aging-associated phenotypes and attenuates cellular apoptosis [182, 224]
		ERRγ (K195)	Promotes ERRγ degradation, thereby reducing *Cyp7a1* expression	Attenuates cholestatic liver injury and fibrosis [251]
		DDB2 (K35/77)	Promotes DDB2 ubiquitination and detachment from DNA lesions	Promotes the process of NER [102]
		Mtf1	Activates Mtf1 for the induction of Mt	Reduces oxidative stress and inflammation by inducing ROS [156]
		SKP2 (K73/77)	Promotes SKP2 nuclear stability, thus increasing Suv39h1 ubiquitination and exclusion from chromatin, enabling H3K9me2 and H3S10p	Promotes expression of the NF-κB inhibitor IκBα to restrict the NF-κB pathway [162]
		NAMPT (K53)	Promotes NAMPT enzymatic activity	Increases cellular NADPH levels to confer cell resistance to oxidative stress damage [27]
		NPM1	Inhibits NPM1 transcriptional activity and the expression of senescent genes	Inhibits cellular senescence [252]
		PKM2 (K433)	Inhibits PKM2-induced STAT3 phosphorylation and activation	Suppresses macrophage polarization towards the proinflammatory M1 phenotype, inhibiting obesity-associated tissue inflammation and metabolic disorders [26, 144]
		EZH2	Promotes EZH2 DNA binding to promote FOXC1 expression	Ameliorates ischemic stroke-induced inflammation [214]
		XBP1s (K257/297)	Promotes XBP1s degradation	Suppresses ER stress [28]
		Caveolin-1	Promotes autophagic degradation of Caveolin-1	Ameliorates hyperglycemia-induced LDL transcytosis across ECs and atherosclerotic progression [171]
		NFATc4	Promotes NFATc4 nuclear export to inhibit BNP expression	Inhibits cardiac hypertrophy [189]
		FoxO1 (K423)	Promotes FoxO1 nuclear export to increase expression of the glucose-sensing genes *Pdx1* and *Glut2* in pancreatic β-cells; inhibits *PCK1* and *G6PC* expression in liver cells	Maintains the GSIS ability of β-cells and deceases gluconeogenesis to maintain glucose metabolic homeostasis [42, 120]
		GCN5 (K549)	Promotes GCN5 activity to acetylate PGC-1α	Reduces hepatic gluconeogenesis [117]
Mono-ADP-ribosyltransferase	Non- histones	SIRT6	May regulate Sirt6 enzymatic activity [31]	NR
		KAP1	Promotes SIRT6-KAP1-HP1α complex formation and heterochromatin packaging to decrease LINE1 expression	Inhibits genomic instability and senescence[33]
		PARP1 (K521)	Activates PARP1 to recruit DNA repair factors	Promotes DNA damage repair [32]
		KDM2A (R2020)	Promotes the displacement of KDM2A from chromatin	Promotes DNA damage repair, ensures replication fidelity [34]
		BAF170 (K312)	Removes H3K27me3 and promotes chromatin accessibility to enhance HO-1 expression	Promotes the clearance of ROS to protect against cellular oxidative stress [35]
Long-chain fatty deacylase	Histones	H3 (K9/18/27/14/36/56/79)	May inhibit gene expression	NR
	Non-histones	TNF-α (K19/20)	Promotes TNF-α secretion	Promotes inflammation [15]
		R-Ras2 (K192/194/196/197)	Suppresses R-Ras2 plasma membrane translocation and activation	Inhibits cell proliferation [38]
Non- catalytic activity	Non- histones	SIRT6-TIP60-GATA4	Recruits TIP60 to acetylate GATA4 at K328/330; in turn, GATA4 inhibits the deacetylase activity of SIRT6 to promote TIP60-induced H3K9 acetylation, increasing expression of the anti-apoptotic gene Bcl-2	Attenuates DOX-induced cardiomyocyte apoptosis [197]
		SIRT6-Sp1-mTOR	Decreases Sp1 transcriptional activity to represses mTOR signaling gene expression	Regulates global cellular protein synthesis to combat cardiac hypertrophy [190]

53BP1: p53-binding protein 1; VSMC: vascular smooth muscle cell; CHD4: chromodomain helicase DNA-binding protein 4; Nkx3.2: NK3 homeobox 2; GATA5: GATA-binding protein 5; TNFSF4: tumour necrosis factor superfamily member 4; IGF: insulin-like growth factor; ERK1/2: extracellular signal-regulated kinase 1/2; WRN: Werner syndrome helicase-nuclease; NKG2D: natural-killer group 2, number D; Txnip: thioredoxin-interacting protein; SNF2H: SWItch/sucrose nonfermentable catalytic subunit SNF2; DSBs: double-strand breaks; DDR: DNA damage response; Nrf2: nuclear factor erythroid-2 related factor 2; HO-1: haem oxygenase-1; SMAD2/3: nuclear translocation of mothers against decapentaplegic homologue 2/3; ERRγ: oestrogen-related receptor γ; Cyp7a1: cholesterol 7 α-hydroxylase; DDB2: DNA damage-binding protein 2; Mtf: metal transcription factor; Mt: metallothionein; SKP2: S phase kinase-associated protein 2; NPM1: nucleophosmin; Suv39h1:suppressor of variegation 3-9 homologue 1; PKM: M2 isoform of pyruvate kinase; STAT3: signal transducer and activator of transcription 3; FOXC1: Forkhead box C1; XBP1s: X-box-binding protein 1 spliced transcription factor; HMGB1: high mobility group box 1; NFATc4: nuclear factor of activated T cell 4; FoxO1: forkhead box protein O1; Pdx1: pancreatic duodenal homeobox 1; Glut2: glucose transporter 2; PCK1: phosphoenolpyruvate carboxykinase; G6PC; glucose-6-phosphatase; GSIS: glucose-stimulated insulin secretion; GCN5: general control nonrepressed protein 5; PGC-1α: peroxisome proliferator-activated receptor-γ coactivator 1-α; KAP1:KRAB-interacting protein 1; PARP1:poly ADP-ribose polymerase 1; LINE1: long interspersed nuclear element 1; KDM2A:lysine-specific demethylase 2; BAF170: BRG/BRM-associated factor (BAF) chromatin remodeler subunit; NRF2: nuclear factor erythroid 2-related factor 2; NR, not reported.

#### 3.1.1 SIRT6 inhibits LINE1-induced genomic damage

**Figure 2.**
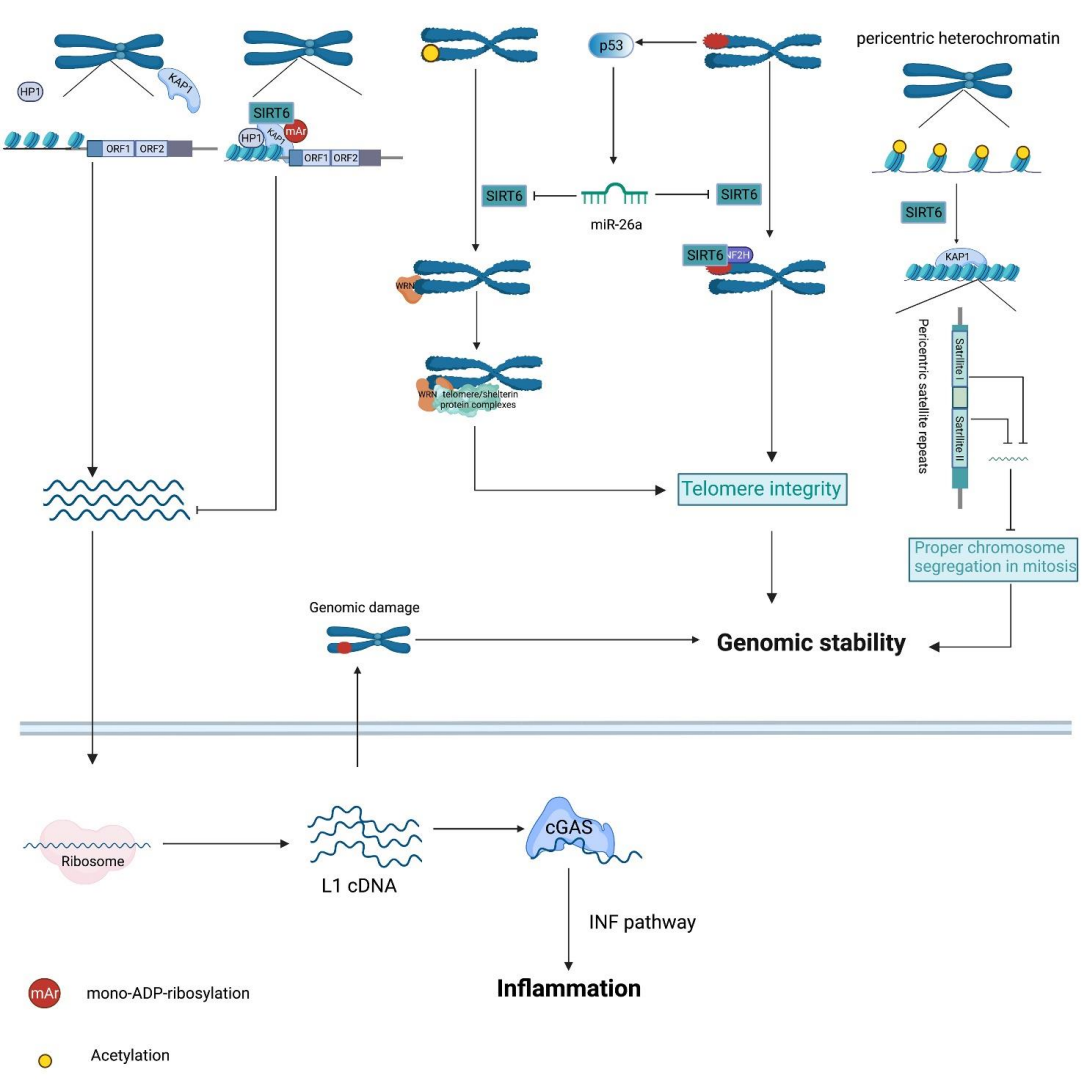
SIRT6 inhibits genomic and telomeric instability. SIRT6 binds to the 5’-untranslated region (UTR) of LINE1 loci and mono-ADP-ribosylates KAP1, which promotes the interaction between KAP1 and HP1α, leading to LINE1 elements packaging into transcriptionally silent heterochromatin. In contrast, loss of SIRT6 in the 5’-UTR of LINE1 elevates LINE1 activity. Accumulation of cytoplasmic LINE1 cDNA instigates chromosomal rearrangements and provokes a strong type I interferon response and subsequent inflammation through the cytoplasmic DNA sensor cGAS. Impaired telomeres upregulate p53 expression, which activates miR-26, thereby decreasing SIRT6 content. During the S phase, SIRT6 deacetylates telomeric H3K9, enabling the efficient binding of the WRN protein to telomeres, leading to the recruitment of telomere/shelterin protein complexes to protect telomere integrity. In response to oxidative stress, SIRT6 recruits SNF2H to damaged telomeres to promote chromatin relaxation, which is associated with the maintenance of telomeres. In addition, SIRT6-mediated deacetylation of H3K18 within pericentric satellite sequences favors the retention of KAP1, repressing satellite transcription and maintaining proper chromosome segregation in mitosis.

Long interspersed nuclear element 1 (LINE1), a retrotransposable element (RTE), is the only RTE capable of autonomous retrotransposition[80]. DNA breaks, insertions and site deletions are required for the replication and integration of LINE1 into host genes, which can cause genomic instability[81]. In response to these threats to genome stability, SIRT6 silences LINE1 retrotransposons through its mono-ADP-ribosyltransferase activity, stabilizing the genome [33] (Fig. 2). Notably, in response to DNA damage and/or during the course of aging, SIRT6 uncouples from LINE1 to enable DNA repair, but which largely compromises the SIRT6-mediated repression of LINE1. The relocalization of SIRT6 is thought to change LINE1 expression patterns and lead to cell dysfunction and aging-related diseases. Therefore, it was unsurprising when studies found that SIRT6-knockout (KO) cells and animals exhibited increased cytoplasmic LINE1 cDNA, which triggered DNA damage, sterile inflammation and pathological progeroid [82]. In addition, pericentric heterochromatin is thought to be associated with proper mitosis and genome stability, and SIRT6-induced H3K18 deacetylation within pericentric satellite sequences has been shown to be involved in the maintenance of pericentric heterochromatin [21, 83].

#### 3.1.2 SIRT6 maintains telomere integrity

Telomeres, complexes with hypoacetylated histone tails, protect linear chromosome ends from DNA degradation to maintain genome integrity. Telomeres undergo shortening, especially during the S phase, and when they reach a critical length, a cell becomes dysfunctional, entering senescence and losing the ability to proliferate [84]. Recent study has revealed that impaired telomeres can repress sirtuin expression, including SIRT6, resulting in further damage to telomere integrity and subsequent progressive deterioration, suggesting SIRT6 is closely associated with telomere integrity maintenance [85] (Fig. 2). SIRT6 deacetylates telomeric H3K9 during the S phase to stabilize the Werner helicase (WRN) in telomeric chromatin and thus prevents aberrant telomere sequence loss [18]. SIRT6 can also maintain telomere-proximal gene silencing, preventing telomere sequence loss and end-to-end chromosomal fusion [76]. In addition, SIRT6 recruits the chromatin remodeling factor SNF2H to decondense chromatin, thereby promoting directional telomere movement, which is associated with the maintenance of telomere integrity [86]. Interestingly, upon exposure to agents that cause DNA damage, SIRT6 deacetylates telomere repeat-binding factor 2 (TRF2) to promote its ubiquitylation and subsequent proteolysis, which may increase the instability of damaged telomeres and promote cell apoptosis [87].

#### 3.1.3 SIRT6 promotes DNA repair

**Figure 3.**
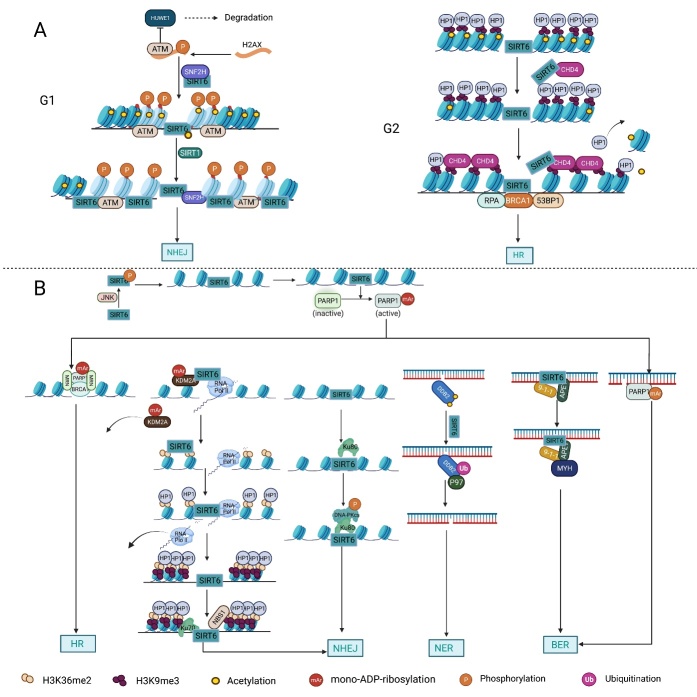
The multitasking roles played by SIRT6 in DNA repair. (A) Chromatin accessibility: In response to DNA damage, both the deacetylation of H3K56 by SIRT6 and the interaction between SIRT6 and SNH2F are important for the recruitment of SNH2F to double-strand break (DSB) sites. The SIRT6/SNF2H complex and ATM cooperatively phosphorylate H2AX at S139 upon DSB formation to block HUWE1-induced H2AX polyubiquitination and degradation, ensuring the efficient formation of γH2AX near damaged sites. γH2AX promotes the retention of DNA repair factors, including ATM, to facilitate nonhomologous end joining (NHEJ) in the G1 phase, and in turn, ATM further phosphorylates H2AX. In addition, SIRT1-mediated deacetylation of SIRT6 at K33 enhances the interaction between SIRT6 and γH2AX. Subsequently, this interaction promotes SIRT6 retention on the chromatin flanking DSBs, enhancing the levels of deacetylation of H3K9 and H3K56 near damaged DNA sites. Similarly, SIRT6-mediated deacetylation of H3K9 and the interaction between SIRT6 and CHD4 both promote the loading of CHD4 onto H3K9me3 to competitively exclude HP1α from chromatin, leading to chromatin relaxation and the recruitment of DNA repair factors to induce efficient HR. (B) DNA repair: Under oxidative stress, SIRT6 is phosphorylated by JNK at S10, which in turn recruits PARP1 to DBSs, and SIRT6 mono-ADP-ribosylates PARP1 at K521. PARP1 mono-ADP-ribosylation is necessary for the efficient recruitment of DNA repair factors such as the MRN complex and BRCA1. SIRT6 directly binds to Ku80 and promotes the interaction between Ku80 and DNA-PKcs, which, in turn, promotes the phosphorylation of DNA-PKcs at S2056 and enhances NHEJ. In addition, SIRT6 mono-ADP-ribosylates KDM2A at R1020 within a leucine rich repeat (LRR), which facilitates displacement of KDM2A from chromatin and the subsequent increase in H3K36 dimethylation near damaged DNA sites. The accumulated H3K36me2 serves as a platform to recruit both early DNA repair components and HP1α; the former promotes NHEJ, and the latter promotes the deposition and spreading of H3K9me3 marks around DSB sites to reduce the abundance of RNA Pol II and thus ensure replication fidelity. SIRT6 deacetylates DDB2 at K35 and K77 to promote DDB2 ubiquitination, which enhances the affinity between DDB2 and ubiquitin-selective p97 segregation and subsequently releases DDB2 from damaged DNA sites. Removal of DDB2 results in relaxation of the nucleosomes around the damaged site and accumulation of downstream DNA repair factors to initiate the nucleotide excision repair (NER) cascade to repair DNA damage. The mono-ADP-ribosylation activity of SIRT6 is necessary for the functional interactions between SIRT6, Rad9-Rad1-Hus1, MYH and APE1, which promote efficient base excision repair (BER). In addition, SIRT6 promotes BER in a PARP1-dependent manner.

The significance of SIRT6 in DNA repair was first suggested by scientists who observed that *Sirt6*-deficient cells exhibited DNA damage hypersensitivity, genomic instability and progeroid-associated degenerative phenotypes, which are also linked to DNA repair and aging [88]. Additionally, in neurodegenerative studies, SIRT6 protein levels were reduced in both Alzheimer's disease patients and mouse models, and overexpression of SIRT6 in hippocampal neurons protected cells from amyloid-beta42 (Aβ42)-induced DNA damage [89]. As research has advanced in recent years, more direct and detailed evidence confirming the roles played by SIRT6 in various DNA repair pathways has been described (Fig. 3).

##### 3.1.3.1 DNA double-strand break (DSB) repair

DNA double-strand breaks (DSBs) are severe DNA lesions that often impair genome integrity [90]. Recent studies have indicated that SIRT6 and DNA DSB repair, but not base excision repair (BER), coevolved with longevity systems, further highlighting the importance of SIRT6 in DSB repair [91]. In response to DSBs, SIRT6 decreases acetylated H3K9 (H3K9Ac) levels and recruits DNA-protein kinase catalytic subunit (DNA-PKcs) to chromatin near damaged DNA sites to promote DNA repair [92]. Several DSB sensors, such as PARP1, Ku 70/80 and the MRE11-RAD50-NBS1 (MRN) complex, have been identified [93, 94]. Recently, SIRT6 has been found to act as a DSB sensor to detect and as a dimer directly bind sites with damaged DNA ends prior to broad cascade activation that initiates the DNA damage response (DDR) [95]. In addition, SIRT6 is also closely associated with the three other aforementioned sensors [32, 96-98]. In response to DNA damage, c-Jun N-terminal kinase (JNK) phosphorylates SIRT6 at serine(S) 10 to promote its mobilization to DNA break sites, which is followed by PARP1 recruitment and efficient DSB repair [99]. At damaged DNA sites, SIRT6 physically interacts with PARP1 and catalyzes its mono-ADP-ribosylation at K521, stimulating PARP1 poly-ADP-ribosyltransferase activity, which promotes DSB repair [32]. In addition, overexpression of SIRT6, but not the individual or combinatory re-expression of homologous recombination (HR)-related proteins, such as NBS1, Rad51, or Rad52, efficiently attenuated the decline in HR in aging cells, and this outcome was dependent on SIRT6 mono-ADP ribosylation, emphasizing the importance of SIRT6 in coordinating these DNA repair proteins [97]. A study of induced pluripotent stem cells (iPSCs) demonstrated that SIRT6 promoted the interaction between Ku80 and DNA-PKcs, thereby facilitating DNA-PKcs auto-phosphorylation and nonhomologous end joining (NHEJ) [96]. Through its mono-ADP-ribosylation of KDM2A, SIRT6 promoted KDM2A detachment from chromatin and subsequently increased the levels of H3K36me2[34]. H3K36me2 serves as a platform for HP1α and DNA repair component accumulation, forming a transcription-silencing microenvironment and promoting NHEJ of transcribed chromatin, respectively [34]. In summary, SIRT6 dexterously averts clashes between transcription and DNA repair to promote efficient DNA repair.

##### 3.1.3.2 Base excision repair (BER) and nucleotide excision repair (NER)

Both BER and NER are involved in DNA repair. SIRT6 improves genome stability by regulating DNA polymerase β (polβ) activity, which influences BER, but the precise mechanism by which SIRT6 affects polβ activity is still unclear [63]. A recent study found that overexpression of SIRT6 attenuated BER deficiency in aging cells in a PARP1-dependent manner [100]. In addition, under conditions of oxidative DNA damage, SIRT6, AP endonuclease 1 (APE1) and the checkpoint clamp Rad9-Rad1-Hus1 (9-1-1) cooperatively bound to the DNA glycosylase MutY homolog (MYH), forming a complex that promoted efficient BER [101]. The mono-ADP ribosylation activity of SIRT6 plays important role in its functional interactions [101]. In addition to BER, SIRT6 deacetylates damaged DNA-binding protein 2 (DDB2) to promote its ubiquitination and separation from chromatin, eventually resulting in relaxation of the nucleosomes around damaged sites to initiate NER [102] (Fig. 3).

#### 3.1.4 SIRT6 modulates chromatin accessibility

The wrapping of eukaryotic DNA into chromatin around nucleosomes leads to an additional physical barrier that prevents access to DNA by DNA repair factors [103]; hence, chromatin relaxation is essential for DDR. It has been established that chromatin remodeling factors and posttranslational modifications of histone proteins, especially acetylation/deacetylation, can significantly affect chromatin structure and promote DNA repair [104, 105]. The interaction between SIRT6 and chromatin remodeling factors was first suggested by scientists who observed that SIRT6-induced H3K56 deacetylation promoted the recruitment of the chromatin remodeler SNF2H to DSBs, which was critical for the proper unfolding of chromatin and subsequent recruitment of DNA repair factors [106]. In addition, the SIRT6/SNF2H complex has been shown to facilitate ATM blocking of HUWE1-mediated H2AX degradation, thereby promoting H2AX stabilization and subsequent recruitment to DSBs [107]. ATM-induced formation of γH2AX foci is important for the retention of DNA repair factors including deacetylated SIRT6 to DSBs, where SIRT6 deacetylates H3K9 and H3K56 to facilitate chromatin remodeling and subsequent DNA repair [59, 107]. Specifically, SIRT6-mediated H3K9 deacetylation facilitated the CHD4 loading at a DNA damage site, where CHD4 competitively excluded HP1α from chromatin to promote chromatin relaxation and HR [108]. Additionally, SIRT6/SNF2H were also essential for NHEJ in the G1 phase, whereas SIRT6/CHD4 were indispensable for HR in the G2 phase [108]. These findings suggest that basis on cell and chromatin status, SIRT6 flexibly regulates the availability of key chromatin remodeling factors to facilitate DSB repair. In general, SIRT6 is involved in modulating chromatin accessibility by either modifying histone proteins or regulating the recruitment of chromatin remodeling factors, enabling cells to respond faster and more efficiently to DSBs (Fig. 3).

### 3.2 SIRT6 maintains metabolic homeostasis

**Figure 4.**
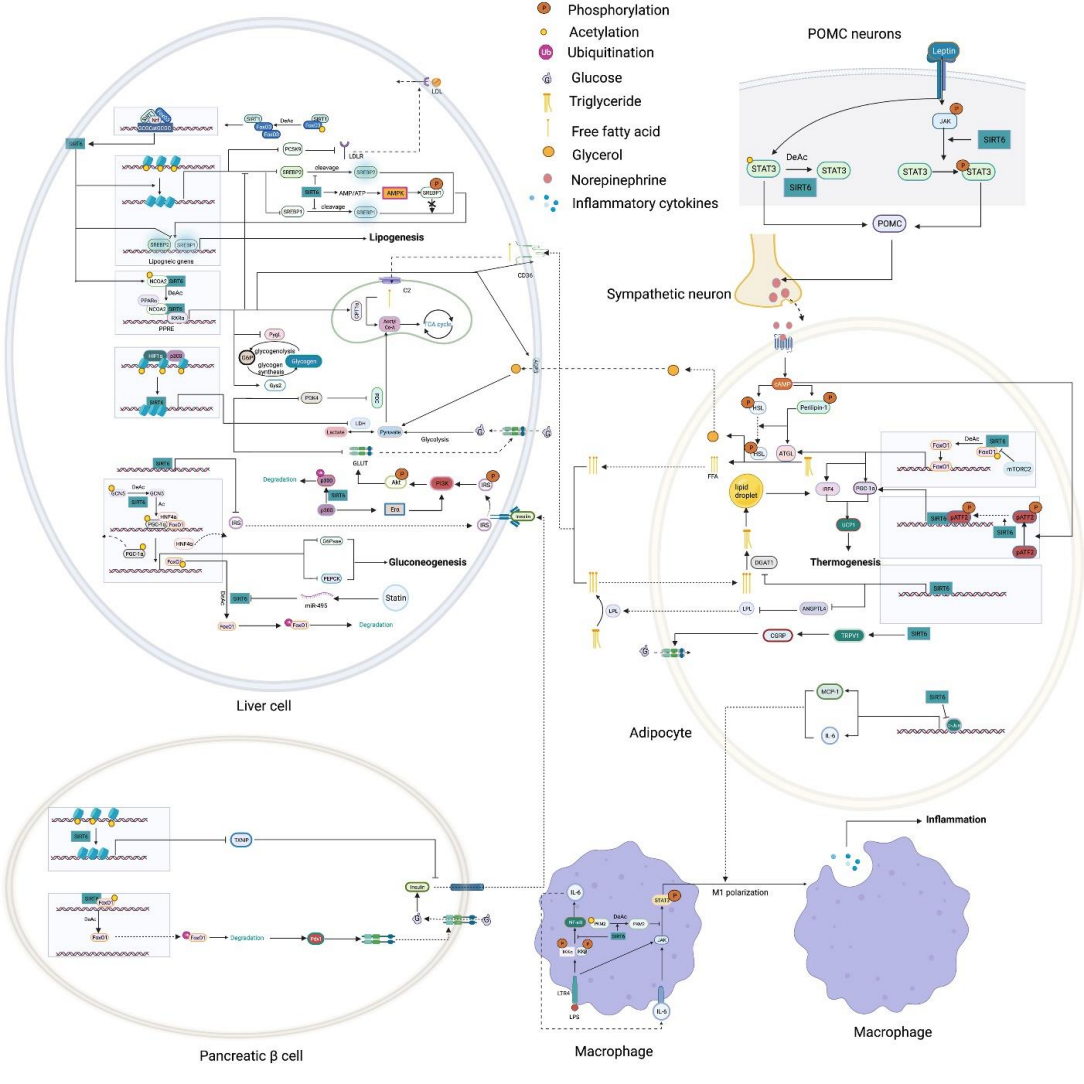
The roles played by SIRT6 in metabolic homeostasis. Glucose metabolism: By deacetylating H3K9 on HIF1α target genes, SIRT6 downregulates PDK4 expression to maintain the catalytic activity of the mitochondrial pyruvate dehydrogenase complex (PDC); LDH suppresses lactate production; and GLUT decreases glucose uptake. SIRT6 also inhibits insulin receptor and insulin receptor substrate (IRS) expression, repressing the phosphorylation of AKT at serine 473 (S473) and threonine 308 (T308) and subsequent glucose uptake. In pancreatic β-cells, SIRT6 inhibits Txnip expression to maintain cell function and survival. In addition, SIRT6-induced FoxO1 deacetylation promotes FoxO1 nuclear export and subsequent degradation, releasing the FoxO1 transcriptional repression of Pdx1 and Glut2. Increased Glut2 and Pdx1 expression promote glucose uptake by β-cells and subsequent insulin production and secretion, increasing glucose uptake and consumption by liver cells. In adipocytes, SIRT6 activates the TRPV1-CGRP-GLUT4 signaling axis to promote glucose uptake. However, in liver cells, SIRT6 downregulates p300 in a ubiquitin-proteasome system-dependent manner to reduce the expression of the estrogen receptor ERα, which restricts PI3K and AKT phosphorylation, eventually disrupting insulin signal transduction and cellular insulin sensitivity. SIRT6 deacetylates GCN5 at K549 to promote its phosphorylation and subsequent acetyltransferase activity on PGC-1α. The acetylation of PGC-1α compromises its ability to promote the expression of gluconeogenic genes, such as PCK1 and G6PC. In addition, statins increase endogenous miR-495 expression to downregulate SIRT6 expression, inhibiting PGC-1α coactivation factor FoxO1 deacetylation and subsequent ubiquitination and degradation. In a PPARα-dependent manner, SIRT6 inhibits Pygl expression to decrease glycogenolysis and promotes Gys2 expression to increase glycogen synthesis. Lipid metabolism: In liver cells, SIRT1 deacetylates FoxO3 to promote the formation of the SIRT1-FOXO3a-NRF1 (SFN) complex on the SIRT6 promoter, increasing SIRT6 protein expression levels. FoxO3 also recruits SIRT6 to the promoter of SREBP2. By deacetylating H3K9 and H3K56, SIRT6 inhibits SREBP1/2 and PCSK9 expression to suppress lipogenic gene expression and low-density lipoprotein (LDL) receptor degradation, respectively. SIRT6 inhibits the cleavage of SREBP1/2 to prevent their activation. Sirt6 also increases the AMP/ATP ratio to promote AMPK-mediated phosphorylation of SREBP1, which inhibits its cleavage. SIRT6 deacetylates NCOA2 at K780 to activate PPARα transcriptional activity. Activated PPARα binds to the retinoic acid receptor RXRα to form a heterodimer that regulates different metabolic pathways. In a PPARα-dependent manner, SIRT6 inhibits SREBP1/2 and their target gene expression to suppress cholesterol biogenesis; stimulates the expression of the FA transporter cluster of differentiation 36 (CD36), acetyl carnitine (C2) and the β-oxidation activator Cpt1α to promote fatty acid utilization; and promotes glycerol transporter Aqp3 expression, leading to increased glycerol uptake. In POMC-expressing neurons, SIRT6 maintains the leptin-induced phosphorylation of STAT3, which promotes POMC production, while SIRT6 can reduce POMC production by deacetylating STAT3. POMC promotes sympathetic activity in adipose tissues, increasing norepinephrine release to increase the cAMP level in adipocytes. An increased cAMP level promotes phosphorylation of hormone-sensitive triglyceride lipase (HSL), Perilipin-1 and ATF2. On the one hand, phosphorylated Perilipin-1 activates ATGL, and on the other hand, it transfers activated HSL from the cytoplasm to the lipid droplet surface, thereby promoting lipolysis. The breakdown of triglycerides yields free fatty acids (FFAs) and glycerol, which are then transported to liver cells through the long-chain FA transporter CD36 and the glycerol transporter aquaporin 3 (AQP3). In adipocytes, SIRT6 promotes deacetylation of FoxO1 to increase ATGL expression. In addition, SIRT6 suppresses DGAT1 expression to inhibit the synthesis of triglycerides. SIRT6 also suppresses the expression of ANGPTL4 to upregulate lipoprotein lipase (LPL) production, thereby enhancing the clearance of serum triglycerides. In brown adipocytes, SIRT6-mediated deacetylation of FoxO1 promotes interferon regulatory factor 4 (IRF4) and PGC1α expression to promote UCP1 expression, thereby enhancing thermogenesis. In addition, SIRT6 promotes p-ATF2 binding to the promoter of PGC1α, upregulating PGC1αexpression. However, SIRT6 suppresses the expression of c-JUN target genes (MCP-1 and IL-6) to inhibit inflammation in adipose tissue. In macrophages, SIRT6 not only inhibits the expression of the NF-KB target gene IL-6 but also prevents STAT3 phosphorylation by deacetylating PKM2 at K433, thereby disrupting the activation of the NF-κB-IL-6-STAT3 axis and preventing macrophage polarization and migration toward adipose tissue.

As an NAD(+)-dependent deacetylase, SIRT6 can regulate various metabolic pathways in response to cellular energy demands [109] (Fig. 4). *Sirt6*-deficient mouse models showed severe metabolic disorders, including elevated insulin resistance, high serum triglyceride levels and obesity, suggesting important roles of SIRT6 in glucose, lipid and energy metabolism [110].

#### 3.2.1 SIRT6 in glucose metabolism

Early studies showed that *Sirt6*-deficient mice presented with lethal hypoglycemia, highlighting the regulatory role played by SIRT6 in glucose metabolism [88]. In-depth studies showed that SIRT6 regulates glucose homeostasis through multiple complex processes, including glycogen synthesis and glycogenolysis, glucose uptake, insulin sensitivity, insulin secretion and gluconeogenesis at the cellular level.

##### 3.2.1.1 Glycolysis and glucose transport

*Sirt6*-deficient mice presented with an intrinsic increase in glucose uptake in both brown adipose tissue (BAT) and muscle [111]. Mechanistically, SIRT6 competed with HIF1α at the promoters of several glycolytic genes, including glucose transporter 1 (GLUT1), pyruvate dehydrogenase kinase 4 (PDK4), and lactate dehydrogenase (LDH), deacetylating histone H3K9 at those promoters, which suppresses the expression of these genes [111]. In addition, SIRT6 deficiency led to the increased membrane association of GLUT1 and GLUT4 and activated the AKT signaling pathway by increasing the expression of insulin receptor substrates (IRSs), such as IRS1 and IRS2, eventually enhancing glucose uptake [112]. However, a separate study reported that overexpression of *Sirt6* increased insulin-stimulated glucose uptake in skeletal muscle and liver, suggesting that SIRT6 engaged in tissue-specific regulation of glycometabolism [113]. Because SIRT6 can lower blood glucose levels by enhancing glycolysis and glucose uptake, SIRT6 inhibition has been considered a promising therapeutic treatment for hyperglycemia. Recently, the application of small-molecule SIRT6 inhibitors has been shown to be a viable strategy for regulating blood glucose levels [114, 115]. These studies with type 2 diabetes mellitus (T2DM) mice model suggested that SIRT6 inhibitors increased oral glucose tolerance by upregulating the expression of GLUT1 and GLUT4 in muscle and enhancing glycolytic activity.

##### 3.2.1.2 Gluconeogenesis

Net hepatic glucose production reflects the net increase in glucose flux due to glycogen synthesis, glycogenolysis, gluconeogenesis, glycolysis and other metabolic activities, and dysregulated gluconeogenesis, not glycogen breakdown, is a primary cause of T2DM [116]. *In vivo*, hepatic SIRT6 overexpression suppressed gluconeogenesis to lower blood glucose levels in diabetic mice model [117]. Mechanistically, SIRT6 deacetylates GCN5 to upregulate its acetyltransferase activity against peroxisome proliferator-activated receptor-γ coactivator 1-α (PGC-1α) [25], thereby reducing the expression of gluconeogenic genes, such as glucose-6-phosphatase (G6PC) and phosphoenolpyruvate carboxykinase (PCK1), and blood glucose levels [116]. In addition, SIRT6-mediated FoxO1 deacetylation promotes its nuclear export, which inhibits G6PC and PCK1 expression to suppress gluconeogenesis [42]. However, some previous studies showed that liver-specific *Sirt6* KO did not affect gluconeogenesis [41], and in contrast, in the context of aging, SIRT6 promoted hepatic gluconeogenic gene expression to maintain energy homeostasis [118].

##### 3.2.1.3 Insulin sensitivity and secretion

Dysregulated insulin secretion and decreased β-cell mass contribute to the development and progression of diabetes mellitus [119]; therefore, maintaining β-cell physiological function and proper insulin secretion are important for the organismal response to changes in blood glucose. *Sirt6* overexpression protected model mice from developing hyperglycemia and decreased glucose tolerance after eating a high-fat diet (HFD) [113]. In contrast, compromised glucose-stimulated insulin secretion (GSIS) has been commonly observed in *Sirt6*-KO pancreatic β-cells [120-122]. These data suggest that the impaired insulin sensitivity and pancreatic β-cell function may cause glucose metabolic disorder in the *Sirt6*-depleted mice. Specifically, SIRT6 can maintain mitochondrial function and regulate Ca^2+^ dynamics, affecting cell membrane depolarization and/or signaling after depolarization, regulating insulin secretion [121]. Furthermore, SIRT6-induced deacetylation of FoxO1 triggers its nuclear export and degradation, which increases pancreatic duodenal homeobox 1 (Pdx1) and glucose transporter 2 (GLUT2) expression, upregulating insulin secretion[120]. In an obese HFD-fed mice model, SIRT6 was found to activate the transient receptor potential vanilloid 1 (TRPV1)-calcitonin gene-related peptide (CGRP)-GLUT4 signaling axis to increase adipocyte glucose uptake and insulin sensitivity [123]. In addition, SIRT6 also cloud downregulate the expression of thioredoxin-interacting protein (Txnip), thereby attenuating glucose-stimulated glucotoxicity, promoting the survival of β-cells and partially increasing glucose tolerance [124]. However, a separate study found that SIRT6 disrupted insulin signal transduction and reduced insulin sensitivity in the liver through the Sirt6-p300-Erα-PI3K signaling pathway, suggesting SIRT6 regulated insulin sensitivity of liver in a gender-dependent manner [125].

##### 3.2.1.4 Diabetic cardiomyopathy

Insulin resistance, hyperinsulinemia and diabetes mellitus are known to induce the development of diabetic cardiomyopathy (DCM) [126]. Inhibition of SIRT6 accelerates pathological processes closely related to DCM, including the inflammatory response and oxidative stress, thereby exacerbating DCM development [127]. A recent study showed that SIRT6 overexpression enabled mitochondria to maintain their functions and protect myocardial cells from DCM development by downregulating the expression of the negative Nrf2 regulator Kelch-like ECH-associated protein 1 (Keap1), which stabilized Nrf2 [128]. In addition, melatonin-mediated activation of the SIRT6-AMPK-PGC1α-AKT axis has been found to attenuate DCM development and progression and subsequently to reduce myocardial vulnerability to myocardial I/R injury [129]. Furthermore, SIRT6 has been shown to attenuate high-glucose-induced apoptosis by activating the AMPK pathway in podocytes [130]. Recent clinical studies showed that metformin treatment led to improved clinical outcomes for acute myocardial infarction patients with prediabetes by improving SIRT6 expression, which reduced inflammatory factor effects [131].

##### 3.2.2 SIRT6 in lipid metabolism

The important roles of SIRT6 in regulating lipid metabolism have been observed in animal models. In hepatic-specific SIRT6 knockout mice, increased triglyceride accumulation, decreased β-oxidation and accelerated fatty liver formation were observed [41]. Additionally, neural-specific *Sirt6*-KO mice did not develop fatal hypoglycemia but presented with growth retardation and eventually became obese [132]. Transgenic mice overexpressing *Sirt6*, however, exhibited reduced accumulation of visceral fat, triglycerides and low-density lipoprotein cholesterol (LDL-C) after consuming a HFD [110].

##### 3.2.2.1 Lipid synthesis

In adipocytes, SIRT6 has been shown to suppress the transcription of specific peroxisome proliferator-activated receptor γ (PPARγ)-regulated genes such as angiopoietin-like protein 4 (ANGPTL4) and diacylglycerol acyltransferase 1 (DGAT1), facilitating the clearance of serum triglycerides and reducing triglyceride synthesis, respectively [110]. SIRT6 also negatively regulates cholesterol biosynthesis in the following three ways: repressing the expression of sterol-regulatory element binding protein 1 and 2 (SREBP1 and SREBP2) and their target genes; restricting the cleavage of SREBP1/SREBP2 to prevent the formation of their active forms; and activating AMPK to inhibit the phosphorylation of SREBP1, eventually inhibiting its nuclear translocation and lipogenic program activation in the liver [133, 134]. In addition, SIRT6 activates PPARα to inhibit SREBP-dependent cholesterol synthesis [135]. Moreover, SIRT6 downregulates the proprotein convertase subtilisin/kexin type 9 (PCSK9) by deacetylating H3K9 and H3K56, preventing LDL receptor degradation and causing increased LDL uptake and decreased serum LDL-C concentrations [136]. In addition, SIRT6 activates the expression of the CDP-diacylglycerol synthase CDS1 and CDS2, promoting the de novo biosynthesis of cardiolipin (CL) and attenuating palmitic acid-induced lipid accumulation [137]. Finally, SIRT6 can remodel the chromatin structure to modulate BMAL1: CLOCK binding to DNA and thus regulate the rhythmic expression of proteins involved in FA and cholesterol metabolism [138]. Therefore, SIRT6 may be an important mediator between metabolic cues and epigenetic signaling to control circadian rhythmicity.

##### 3.2.2.2 Lipolysis

Previous studies showed that SIRT6 increased β-oxidation by activating AMPK [45, 139]. Recently, SIRT6 has been found to deacetylate the PPARα coactivator NCOA2 to upregulate PPARα activity and the expression of its target gene carnitine palmitoyl transferase 1a (Cpt1a), eventually increasing lipid consumption [135]. A separate study found that PKCζ phosphorylated SIRT6 to promote its enrichment at the promoters of FA β-oxidation-related genes, inducing their expression [58]. In addition, SIRT6-induced deacetylation of FoxO1 increased its nuclear retention and transcriptional activity to induce the expression of the key lipolytic enzyme adipose triglyceride lipase (ATGL) to upregulate lipolysis [140]. In pro-opiomelanocortin (POMC)-expressing neurons, SIRT6 ensured activation of the leptin-induced JAK-STAT3 signaling cascade, increasing POMC production and thus promoting sympathetic nervous system (SNS) activity, which was closely related to the lipolytic activity and browning of adipose tissue [141]. Unexpectedly, SIRT6 overexpression has been shown to reduce STAT3 acetylation, which reduced POMC production, impairing SNS activity in adipose tissue [142]. These findings indicate the importance of maintaining SIRT6 activity within a physiological range.

##### 3.2.2.3 Immunoreactions in adipose tissue

An association between continued inflammation of adipose tissue and metabolic pathway disorders has been established, making inflammatory pathways appealing targets in novel treatments of metabolic diseases and their related complications [143]. Fat-specific *Sirt6* ablation increased the expression of two proinflammatory cytokines MCP-1 and IL-6 [140]. Furthermore, myeloid *Sirt6* deficiency led to obesity-associated tissue inflammation and subsequent insulin resistance and macrophage infiltration[144]. Upon lipopolysaccharide (LPS) stimulation, activation of NF-κB and endogenous IL-6 production were promoted, which resulted in STAT3 activation and positive feedback signaling that stimulated NF-κB expression [144]. SIRT6 has been shown to deacetylate nuclear PKM2, leading to inhibited STAT3 phosphorylation, which disrupted NF-κB-IL-6-STAT3 axis activation, inhibiting the migration of macrophages to adipose tissue and macrophage polarization toward the proinflammatory M1 phenotype [26, 144]. These findings suggest that SIRT6 plays a protective role in controlling adipose tissue inflammation.

##### 3.2.2.4 Thermogenesis in adipose tissue

Nonshivering thermogenesis in BAT substantially increases energy expenditure, which makes it a promising therapeutic target for the treatment of obesity and associated diseases [145]. Recently, SIRT6 has been shown to upregulate thermogenesis in response to cold and increase β-adrenergic agonist effects in BAT [146]. SIRT6 is an adaptor protein that recruits the phosphorylated activating transcription factor 2 (p-ATF2) protein to upregulate the expression of *PGC-1α* and the downstream thermogenic gene *UCP1* [146]. However, *Sirt6*-KO mice showed insulin resistance, which corresponded with decreased expression of adiponectin in white adipose tissue (WAT) and *UCP1* in BAT and increased inflammatory signaling in both WAT and BAT [147]. Similarly, SIRT6 could promote *UCP1* and interferon regulatory factor 4 (*IRF4*) expression by deacetylating FoxO1 in BAT [56]. Hence, SIRT6 has the capacity to promote nonshivering thermogenesis mediated by UCP1, which may be a promising target for treatments that maintain metabolic homeostasis.

### 3.3 SIRT6 regulates oxidative stress and inflammation

#### 3.3.1 SIRT6 alleviates oxidative stress

Oxidative stress, characterized by an imbalance between ROS production and clearance, is an important pathological mechanism that causes disease [148]. Previous studies have proven that oxidative stress can lead to downregulated SIRT6 activity [149, 150], and conversely, SIRT6 can alleviate oxidative stress through multiple signaling pathways, among which the SIRT6-NRF2-HO-1 pathway is the best characterized [151-153]. Specifically, SIRT6 deacetylates H3K56 at the HO-1 promoter, triggering the assembly of the NRF2-RNAP II transcription complex and upregulating HO-1 expression [154]. Interestingly, these observations seem to contradict findings indicating that SIRT6 interacts with RNA Pol II and negatively regulates the release of negative elongation factor (NELF) to stabilize RNA Pol II promoter-proximal pausing, thereby inhibiting gene expression [155]. Recently, additional research revealed that SIRT6 promotes the transcription of NRF2 target genes through its mono-ADP-ribosyltransferase activity but not deacetylase activity, and in contrast, SIRT6-induced deacetylation of H3K56 may suppress the NRF2 response [35]. Under oxidative stress conditions, SIRT6 catalyzes the mono-ADP-ribosylation of BRG/BRM-associated factor 170 (BAF170), promoting its recruitment to the E2 enhancer at the HO-1 promoter to promote chromatin remodeling. Subsequently, chromatin remodeling reduces chromain physical barrier for recruitment of RNA Pol II and NRF2. In addition, SIRT6 can deacetylate metal transcription factor (Mtf), a key transcription factor of metallothionein (Mt) 1 and 2, to counteract oxidative stress [156]. SIRT6 also directly deacetylates NAMPT to increase cellular NAD^+^ and NADPH levels and protect against oxidative stress [27].

#### 3.3.2 Anti-inflammatory and proinflammatory effects of SIRT6

Inflammation is a complex pathophysiological response to damage; in general, the inflammatory cascade results in tissue damage and organ dysfunction [157]. Among proinflammatory cytokines, NF-κB and TNF-α are two important modulators of inflammation-related pathways. Recent studies found that the important roles played by SIRT6 in regulating anti-inflammatory responses were largely dependent on the regulated expression or protein functions of TNF-α and NF-κB. In macrophages, SIRT6 repressed NF-κB and endogenous IL-6 expression, thereby disrupting the crosstalk between NF-κB and STAT3 and inhibiting macrophage polarization toward the proinflammatory phenotype [144]. In myocardial I/R, SIRT6 restricted the NF-κB pathway to attenuate mitochondrial defects and cell death [158]. Under pressure overload, SIRT6 inhibited the expression of regulators downstream of NF-κB to attenuate inflammation and cardiac fibrosis[159]. Mechanistically, SIRT6 blocks the NF-κB signaling pathway at multiple levels. First, SIRT6 inhibits NF-κB-targeted gene expression by deacetylating H3K9 [160]. Second, SIRT6 restricts the transcriptional activity of the NF-κB subunit RelA via deacetylation [161]. Finally, SIRT6 accelerates negative feedback loop signaling that downregulates NF-κB activity by enhancing the expression of IκBα, an important inhibitor of NF-κB [162].

**Figure 5.**
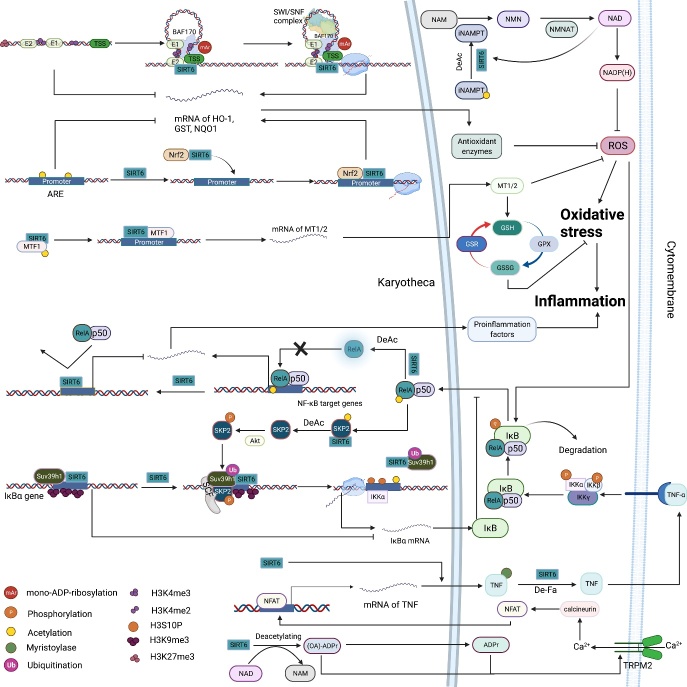
The roles played by SIRT6 in the regulation of oxidative stress and inflammation. (A) Oxidative stress: SIRT6 deacetylates H3K56 at the promoter of Nrf2 target genes and is a bridge that recruits the NRF2-RNAP II transcription complex, thereby upregulating antioxidant enzyme expression. Furthermore, in response to oxidative stress, SIRT6 mono-ADP-ribosylates BAF170 at K312 to promote the recruitment of the SWI/SNF complex to the promoter region of HO-1, facilitating chromatin loop formation at the HO-1 locus to reduce the total physical volume between the enhancer and transcription start site. On the promoter of Mt1/2, SIRT6 physically interacts with and deacetylates Mtf1, coactivating the transcription of Mt1 and Mt2 to boost cell defenses against reactive oxygen species (ROS). Moreover, increased Mt1 and Mt2 levels promote the GSH-GSSH system to counteract oxidative stress. SIRT6 deacetylates NAMPT at K53 to promote its activity, increasing cellular NAD+ and NADPH levels, which confers cell resistance against oxidative stress damage. Increased NAD+ levels also upregulate SIRT6 deacetylase activity. (B) Inflammation: Through deacetylating H3K9 on the promoter of NF-κB target genes, SIRT6 prevents the binding of RelA to chromatin, repressing proinflammatory factor expression. In addition, SIRT6 directly interacts with and deacetylates RelA at K310, which restricts its DNA binding activity, terminating NF-κB signaling. Under TNF-α stimulation, IκBα is phosphorylated, subsequently ubiquitinated and eventually degraded, which leads to the translocation of NF-κB (RelA/p50) to the nucleus, where it binds to target gene promoters and regulates their expression. Moreover, TNF-α-induced activation of NF-κB activates SIRT6, which in turn deacetylates the E3 ubiquitin ligase SKP2 at K73 and K77, resulting in subsequent phosphorylation of SKP2. These modifications enhance the stability and nuclear content of SKP2, contributing to SKP2-mediated monoubiquitination of Suv39h1 and subsequent Suv39h1 release from the promoter of IκBα. Then, the H3K9 demethylation rate is increased, and H3S10 is phosphorylated by IKKα on the IκBα promoter, eventually promoting the transcription of IκBα. The product of SIRT6-mediated deacetylation OAADPr and its derivative ADPr activate TRPM2 to promote Ca2+ influx, thereby promoting TNF-α and IL-8 expression through the calcineurin-nuclear factor of activated T cells (NFAT) pathway. SIRT6 promotes TNF-α secretion by removing the fatty acyl groups from K19/20. SIRT6 upregulates TNF-α mRNA translation efficiency.

Interestingly, however, SIRT6 has also been shown to accelerate the production and secretion of the inflammatory cytokine TNF-α [15, 163, 164]. At the transcriptional level, O-acetyl-ADP ribose (OAADPr), a product of SIRT6-mediated deacetylation, or its derivative ADP ribose (ADPr) can activate transient receptor potential cation channel subfamily M, member 2 (TRPM2) to accelerate Ca^2+^ influx, thereby enhancing the expression of TNF-α and IL-8 [164]. Moreover, studies showed that a high intracellular NAD^+^ concentration can activate immune cells and promote TNF-α synthesis, but SIRT6 is the only sirtuin family member that has been shown to upregulate *TNF-α* mRNA translational efficiency [163]. In addition, upon LPS stimulation, SIRT6 is rapidly localized to the ER and promotes TNF-α secretion through its demyristoylase activit y[15, 165]. Overall, SIRT6 positively regulates TNF-α production and secretion at multiple levels, including at the transcriptional and translational levels and through posttranscriptional modification, demonstrating the important roles of SIRT6 in the transduction of TNF-α signaling. However, there is currently no evidence that SIRT6 exacerbates inflammatory diseases (e.g., atherosclerosis) through promoting TNF-αsecretion.

In summary, in a variety of tissue-derived cells, SIRT6 regulates oxidative stress and inflammation through multiple pathways, especially the NRF2, NF-κB and TNF-α signaling pathways (Fig. 5). Notably, discrepancies between the anti-inflammatory and proinflammatory roles played by SIRT6 may contribute to the specific effects of a cell type and stimulus; for example, SIRT6 deficiency did not influence NF-κB signaling in embryonic stem (ES) cells [111]. In summary, SIRT6 acts as a mediator in the TNF-α-NF-κB signaling axis to maintain proper reaction intensity, which is important for maintaining organismal homeostasis, to some extent. Regardless, the multifaceted roles played by SIRT6 in oxidative stress and inflammation make targeting SIRT6 a reasonable therapeutic strategy.

## 4. SIRT6 regulates the pathophysiology of CVDs

**Figure 6.**
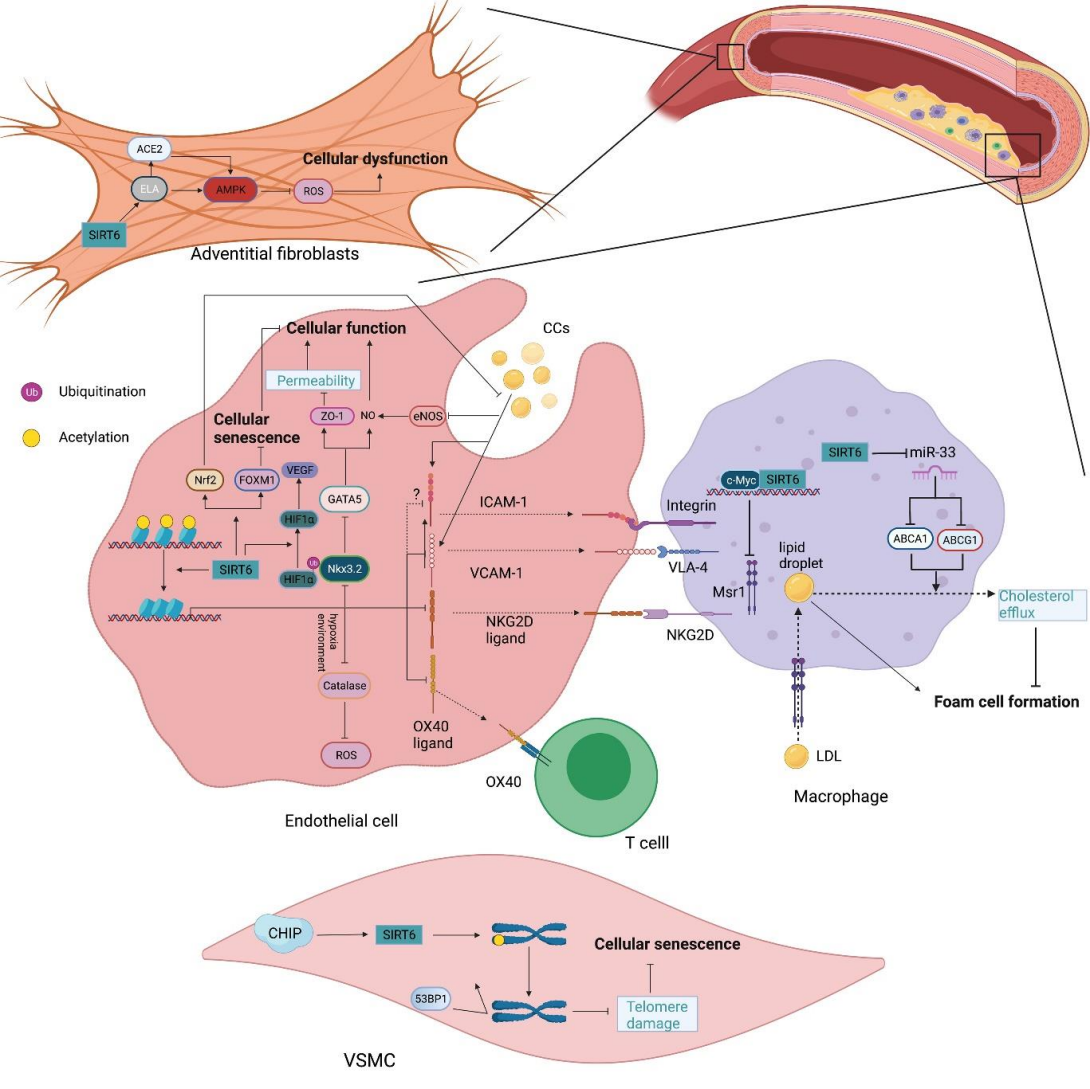
The roles played by SIRT6 in vascular atherosclerosis. In endothelial cells (ECs), SIRT6 downregulates the expression of proinflammatory cytokines, including VCAM-1, ICAM-1, NKG2D ligand and OX40 ligand, by deacetylating H3K9 on their respective promoters, reducing the number of inflammatory cells to inhibit vascular inflammation. SIRT6 also inhibits the cholesterol crystal-induced high expression of ICAM-1 and VCAM-1 by activating Nrf2. In addition, by deacetylating H3K9, SIRT6 suppresses Nkx3.2 expression to promote GATA5, which induces zonula occluden-1 (ZO-1) and NO activity, thereby decreasing cellular permeability and maintaining endothelial cell function. SIRT6 upregulates FOXM1 expression to prevent cellular senescence. Under hypoxic stress, SIRT6 deubiquitinates HIF-1α at K37 and K532 to protect it from decomposition, thus upregulating VEGF expression, which increases angiogenesis. In addition, SIRT6 inhibits catalase activity by deacetylating H3K56 on its promoter, which inhibits reactive oxygen species (ROS) clearance, further aggravating injury and hemorrhage of the neovasculature. In macrophages, SIRT6 downregulates Msr1 expression by suppressing c-Myc transcriptional activity and reducing oxidized low-density lipoprotein (ox-LDL) uptake and foam cell formation. SIRT6 also upregulates ABCA1 and ABCG1 expression by inhibiting miR-33 production and promoting cholesterol efflux. In vascular smoot muscle cells (VSMCs), SIRT6 maintains telomere stability by deacetylating H3K9 to prevent 53BP1 binding, thereby delaying cellular senescence and attenuating atherosclerosis development. In adventitial fibroblasts, SIRT6-ELA-ACE2 signaling can upregulate AMPK activity to reduce the ROS levels, thus protecting against oxidative stress and apoptosis.

**Figure 7.**
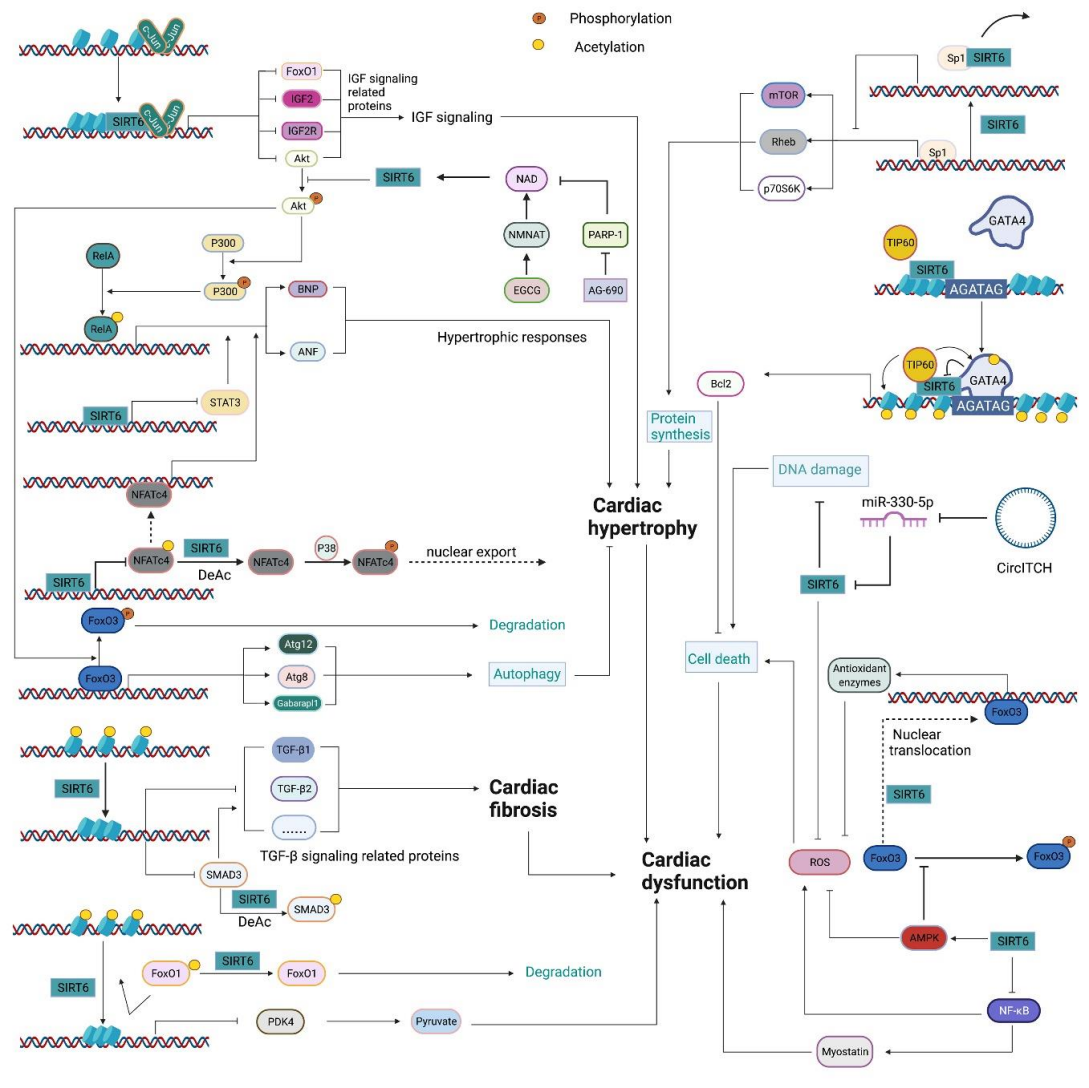
The roles played by SIRT6 in myocardial diseases. At c-Jun target gene promoters, SIRT6 interacts with c-Jun and deacetylates H3K9 to inhibit the expression of IGF signaling-related genes downstream of C-Jun, such as FoxO1, IGF2, IGF2R and Akt, attenuating cardiac hypertrophy and heart failure. SIRT6 also inhibits the phosphorylation of Akt to suppress its activation. Phosphorylated Akt promotes p300 phosphorylation and subsequent expression of NF-κB target genes, such as BNP and ANF, leading to the development of myocardial hypertrophy. In addition, phosphorylated Akt facilitates phosphorylation of FoxO3 and its subsequent nuclear export to inhibit autophagic gene expression, including Atg8, Atg12 and Gabarapl1, promoting pathological growth of cardiomyocytes. EGCG has been shown to upregulate SIRT6 activity by enhancing NMNAT activity and subsequently increasing NAD+ levels. In addition, the novel PARP-1 inhibitor AG-690 inhibits PARP-1 activity to maintain NAD+ intracellular levels, thereby upregulating SIRT6 activity. SIRT6 inhibits the expression and transcriptional activity STAT3 to hinder the expression of its target genes BNP and ANF. In addition, SIRT6 both inhibits NFATc4 expression and deacetylates NFATc4 to promote its nuclear export and subsequent degradation, suppressing the expression of its downstream genes BNP and ANF. On the promoters of mTOR signaling genes, SIRT6 interacts with Sp1 to repress the expression of related proteins, including mTOR, Rheb and p70S6K, thereby inhibiting both the synthesis of abnormal proteins and development of myocardial hypertrophy. In the promoter of Bcl-2, SIRT6 initially occupies this region via its property of high nucleosome-binding affinity and recruits TIP60. GATA4 recognizes the GATA sequence and subsequently interacts with SIRT6 via its C-terminal Zn-finger to form the SIRT6-TIP60-GATA4 complex. In this complex, SIRT6 deacetylase activity is repressed by GATA4, while TIP60 enhances GATA4 transcriptional activity and the acetylation level of local histones, ensuring the transcription of Blc-2. CircITCH sponges miR-330-5p to upregulate SIRT6 expression, attenuating reactive oxygen species (ROS) formation, DNA damage and cardiotoxicity. By deacetylating H3K9 and H3K56, SIRT6 inhibits the expression of TGF-β signaling-related proteins, such as SMAD3, TGF-β1 and TGF-β2. In addition, SIRT6 deacetylates SMAD3 at K333 and K378 to repress its transcriptional activity. SIRT6 deacetylates FoxO1 to promote its degradation, and SIRT6-mediated deacetylation of H3K9 within the PDK4 promoter also suppresses FoxO1 binding to this region, thereby reducing PDK4 expression and the subsequent accumulation of pyruvate and maintaining cardiac function. SIRT6 also inhibits myostatin expression to prevent the development of heart failure and its complications. After cardiac ischemia/reperfusion (I/R), SIRT6 upregulates AMPK activity and downregulates NF-κB signaling pathway activation, reducing cellular ROS levels and attenuating myocardial apoptosis. In addition, SIRT6 inhibits FoxO3 phosphorylation in an AMPK-dependent manner, and SIRT6 promotes FoxO3 nuclear translocation to induce FoxO3 target gene expression and reduce ROS production.

Among aging-related diseases, CVDs are thought to be the most important cause of human death. CVDs arise from cellular senescence, metabolic disorder, inflammation, cell death and aberrant cell growth, which can lead to the dysfunction of blood vessels and pathological cardiac remodeling in the heart. Sirtuins are extensively involved in these processes, regulating the pathophysiological conditions of the heart[166]. Here, we will focus on the roles of SIRT6 in atherosclerosis (early events, Fig. 6), cardiac hypertrophy and fibrosis (middle-stage events), heart failure (end-stage events) and I/R injury (recovery-stage events) in CVDs (Fig. 7).

### 4.1 Atherosclerosis

Endothelial dysfunction promotes the initiation and development of atherosclerosis and its complications [167]. As a guardian that maintains genome stability, SIRT6 can protect endothelial cell (EC) from DNA and telomere damage and thus prevent premature senescence and maintain normal EC functions [79]. In addition, endothelial SIRT6 deficiency induces EC senescence associated with decreased FOXM1 expression [168]. Furthermore, SIRT6 attenuates minute cholesterol crystal (CC)-induced EC dysfunction through Nrf2 activation [169]. By deacetylating histone H3K9, SIRT6 downregulates NK3 homeobox 2 (Nkx3.2) transcription to promote GATA-binding protein 5 (GATA5) expression, thereby increasing the production of vascular endothelial nitric oxide synthase (eNOS) and preventing endothelial injury [66, 170]. SIRT6-induced deacetylation of caveolin-1 contributes to its autophagic degradation, retarding LDL transcytosis across ECs and atherosclerosis progression [171]. In VSMCs, the deacetylase activity of SIRT6 is crucial for delaying cellular senescence and inflammation because it prevents telomere damage, thereby stabilizing atherosclerotic plaque [78].

Atherosclerosis is an inflammatory disease of the arterial intima in which inflammatory cells and inflammatory responses play crucial roles [172]. Endothelial SIRT6 deficiency results in the increased expression of proinflammatory proteins, such as IL-1b and NF-κB [65]. In addition, SIRT6 inhibits the adhesion of monocytes to ECs by decreasing the expression of vascular cell adhesion molecule-1 (VCAM-1) and intracellular adhesion molecule-1(ICAM-1) to relieve atherosclerosis [173]. Additionally, the expression of tumor necrosis factor superfamily member 4 (TNFSF4, also known as OX40 L), a risk factor for atherosclerosis, was reduced by SIRT6 under normal conditions or after TNF-α stimulation [67]. The OX40L and OX40 interaction has been implicated in the activation of T cells and B cells, as well as cytokine production; therefore, SIRT6 may ameliorate atherosclerotic lesions [174]. In addition, SIRT6-induced macrophage autophagy in oxidized LDL-C (ox-LDL-C)-treated ECs increases cholesterol efflux and reduces macrophage infiltration, endothelial inflammation and foam cell formation, thereby attenuating the pathological development of atherosclerosis [175, 176].

As mentioned above, SIRT6 has been shown to accelerate the clearance of serum triglycerides and LDL-C [110, 136]; therefore, SIRT6 exerts a protective effect against atherosclerosis through the maintenance of lipid homeostasis. In contrast, ITCH ubiquitinates SIRT6, promoting its degradation, thereby disrupting FA metabolism and leading to hypercholesterolemia and hyperlipidemia and accelerating atherosclerosis progression [50]. In addition, oxidized LDL (ox-LDL) and its ligand, a scavenger receptor, contribute to the pathogenesis of atherosclerosis [177]. SIRT6 can suppress the expression of miR-33 to upregulate ABCA1 and ABCG1 expression, thereby promoting cholesterol efflux and attenuating atherosclerosis [176]. A separate study showed that SIRT6 was a corepressor of c-MYC expression, which led to the inhibition of macrophage scavenger receptor 1 (Msr1) expression, reducing ox-LDL uptake and inhibiting foam cell formation [178]. By deacetylating H3K9 and H3K56, SIRT6 also suppressed the expression of the ligand NKG2D, inhibiting immune cell recruitment and stabilizing atherosclerotic plaques [179, 180].

Interestingly, however, a separate innovative study showed a pernicious roles of SIRT6 in the development of hemorrhage in unstable carotid plaques [72]. SIRT6 deubiquitinated HIF-1α, protecting it from decomposition, and through its stabilization, HIF-1α upregulated the expression of vascular endothelial growth factor (VEGF) and promoted subsequent angiogenesis under hypoxic stress [72]. Moreover, SIRT6 deacetylated H3K56, downregulating the expression of the ROS scavenger Catalase, which aggravated the accumulation of ROS and subsequent neovascular injury and hemorrhage [72]. SIRT6 induces continuous angiogenesis, and hemorrhage of the neovasculature contributes to the instability of atherosclerotic plaques. In general, SIRT6 exerts an antiatherosclerotic effect, but the adverse effects of SIRT6 overexpression on atherosclerotic plaques are notable. In addition, these findings provide guidance for the development and use of SIRT6 modulators based on the different stages of diseases.

### 4.2 Cardiac hypertrophy and fibrosis

The heart undergoes hypertrophy in response to pressure-overload stimuli, but this hypertrophy generally progresses to heart failure through pathological cardiac remodeling [181]. As an anti-senescence molecule, SIRT6 was shown to attenuate cardiac aging and aging-associated cardiac hypertrophy [182]. In addition, cardiac-specific deletion of *Sirt6* significantly increased left ventricular wall thickness, interstitial fibrosis and cardiomyocyte cross-sectional area, whereas overexpression of *Sirt6* alleviates pressure-overload-induced cardiac hypertrophy and cardiac abnormalities [68, 183]. Indeed, SIRT6 serves as a negative regulator of IGF-Akt signaling, whose constitutive activation ultimately contributes to cardiac hypertrophy [68]. Additionally, SIRT6 reduces Akt protein levels to promote FoxO3 dephosphorylation and nuclear retention, inducing autophagic gene expression and attenuating isoproterenol (ISO)-induced hypertrophy [184]. Inhibition of NF-κB activation could inhibit or even reverse pressure-overload-induced cardiac remodeling and hypertrophy [181]. At the pharmaceutical level, epigallocatechin-3-gallate (EGCG) blocked NF-κB DNA-binding activity to protect against cardiac hypertrophy mediated through the PSMB5-Nmnat2-SIRT6 axis [185, 186]. Furthermore, AG-690/11026014 (6014), a novel PARP-1 inhibitor, has been shown to block Ang II-induced cardiac hypertrophy, which was partially attributed to activation of SIRT6 deacetylase activity through upregulating the intracellular NAD^+^ levels [187]. Furthermore, SIRT6 inhibits phenylephrine (PE)-induced induction of the fetal gene programme through inhibiting STAT3 expression and phosphorylation [188]. Similarly, SIRT6 downregulated the expression of NFATc4 and deacetylated NFATc4 to promote its nuclear export, thereby reducing BNP expression and protecting against cardiac hypertrophy [189]. Cardiac hypertrophy requires an increased rate of protein synthesis, and mTOR signaling controls protein synthesis and degradation [181]. At promoters of mTOR signaling genes, SIRT6 interacted with Sp1 to repress mTOR-related gene expression and subsequent protein synthesis, protecting against abnormal enlargement of cardiomyocyte [190].

Cardiac fibrosis, characterized by the transformation of fibroblasts into myofibroblasts, aggravates the development of pathologic cardiac remodeling and heart failure, and TGF-β/AMSD3 signaling is the key pathway in fibrogenesis [191, 192]. By deacetylating H3K9 and H3K56, SIRT6 suppresses the expression of TGF-β/SMAD3 signaling pathway genes [193]. In addition, SIRT6 can deacetylate SMAD3 to decrease its DNA-binding affinity [193]. Additionally, SIRT6 was reported to inhibit the phenotypic conversion of cardiac fibroblasts to myofibroblasts through blocking NF-κB signaling, thereby inhibiting cardiac fibrosis [69].

### 4.3 Heart failure

The metabolic network of cardiomyocytes contains a complex set of interacting pathways. Disordered energy metabolism in cardiomyocytes, such as a decrease in mitochondrial oxidative capacity, abnormal accumulation of substrate and dysfunctional intermediate utilization, are thought to be important pathogenic mechanisms in heart failure [194]. In *Sirt6*-null cells, HIF1-α expression was upregulated, which increased the expression of glycolytic genes and diminished mitochondrial respiration to shift glucose metabolism toward glycolysis [111]. Intriguingly, however, later research found no noticeable upregulation of HIF-1α-targeted glycolytic gene expression or glucose uptake in *Sirt6*-KO hearts, suggesting that SIRT6 deficiency-induced alterations of systemic glucose homeostasis in cardiomyocytes was not dependent on HIF-1α [68]. Recent findings proved that impaired mitochondrial oxidation in *Sirt6*-KO hearts was mediated by FoxO1 but not HIF-1α [70]. In cardiomyocytes, SIRT6 inhibited PDK4 expression by repressing the FoxO1 occupancy at its promoter, which contributed to enhanced PDH1 activity and subsequent metabolic shifting toward increased glucose oxidation to meet cellular energy requirements [70]. Hence, SIRT6 is indispensable for producing glycolytic substrates in the mitochondria for cardiac oxidation/utilization, and is, at least partially, critical for heart failure in cardiac-specific *Sirt6*-KO mice. In addition, SIRT6 attenuated myocardial lipid accumulation and the development of cardiac dysfunctions by reducing fatty acid uptake [195]. Mechanistically, SIRT6 inhibited the transcriptional activity of PPARγ to negatively regulate the expression of several critical fatty acid transporters, such as caveolin-1 and CD36 [195]. In summary, these studies showed that SIRT6 maintains the cellular energy supply by regulating glucose oxidation and lipid metabolism, thereby enhancing cardiac functions.

In addition to regulating metabolism, SIRT6 also maintains cardiac functions in other ways. For example, SIRT6 inhibits myostatin expression by mitigating NF-κB binding to the myostatin promoter, which is beneficial for maintaining muscle function and preventing chronic heart failure-induced cachexia [196]. In addition, the circular RNA ITCH (CircITCH) has been shown to prevent miR-330-5p-mediated degradation of SIRT6 to ameliorate doxorubicin-induced cardiotoxicity (DOXIC) [47]. Similarly, the SIRT6-TIP60-GATA4 axis couples gene transcription and epigenetic activation, protecting against DOXIC by regulating anti-apoptotic pathway signaling [197]. The nonenzymatic functions of SIRT6 in maintaining cardiac function provide a new perspective for better understanding the significance of SIRT6 in CVDs.

### 4.4 Cardiac ischemia/reperfusion (I/R) injury

The protective roles of SIRT6 in I/R injury have been established. For example, SIRT6 has been found to attenuate cerebral I/R by activating NRF2 signaling to reduce oxidative stress; in contrast, miR-370-induced inhibition of SIRT6 expression aggravates cerebral I/R by affecting the NRF2/ARE signaling pathway [198, 199]. A recent study found that EC-specific depletion of *Sirt6* increased cell death after I/R injury and impaired blood-brain barrier integrity by activating caspase-3 [200]. In addition, cardiomyocytes overexpressing SIRT6 exhibited higher tolerance to hypoxia via reducing ROS production [201]. Subsequent findings showed that SIRT6 could attenuate cellular oxidative stress, myocardial damage, and ventricular remodeling in mice with myocardial I/R injury [71]. Specifically, I/R stress significantly increased the SIRT6 level in the cytoplasm to enhance AMPK activity through upregulating AMP/ATP and then promoting the transcriptional activity of FoxO3α. Subsequently, FoxO3α initiates the expression of antioxidant-encoding genes, such as catalase and superoxide dismutase (SOD2) to accelerate the clearance of ROS and impede cellular apoptosis, improving function and survival of cardiomyocytes in the ischemic heart. In a rabbit model, massive cardiomyocyte apoptosis was observed after myocardial I/R, and even a small increase in the apoptosis rate was considered sufficient to cause heart failure [202]. Hence, SIRT6-inhibited myocardial cell death is crucial for the maintenance of cardiac function after I/R.

Interestingly, silencing *SIRT6* expression and inhibiting NAMPT activity reduced the biosynthesis of the neutrophil chemoattractant CXCL8 in mononuclear cells, thereby attenuating neutrophil infiltration and limiting myocardial infarct size during reperfusion [73]. In view of the protective effects of SIRT6 on myocardial I/R described above, it is surprising that SIRT6 promotes neutrophil infiltration during myocardial I/R. Notably, the authors of the previous study observed infarct size and neutrophil accumulation within 24 h of reperfusion, but long-term cardiac function was not evaluated in this study. New evidence has also shown that neutrophil infiltration is critical for tissue repair because it promotes debris clearance, cytokine scavenging, endothelial recovery, and angiogenesis [203]. Therefore, the potential effects of silencing SIRT6 expression on cardiac function preservation over long periods deserve further observation.

## 5. SIRT6 modulators

Given the important roles of SIRT6 in CVDs and multiple SIRT6-related molecular pathways, including pathways involved in maintaining genome stability and in inflammation and metabolism, regulation of SIRT6 expression and enzymatic activities may provide therapeutic benefits. In recent years, on the basis of details on the SIRT6 structural features, an increasing number of SIRT6 regulatory compounds with finely tuned regulatory effects on SIRT6 and related signaling pathways have been identified [204].

### 5.1 SIRT6 activators

First, some natural plant extracts have been proven to modulate SIRT6 expression or activity. For example, icariin and quercetin, both polyphenols, are known SIRT1 activators [205, 206], and recent studies have shown that they both stimulate SIRT6 deacetylation activity in a dose-dependent manner [198, 207]. In addition, quercetin inhibits SIRT6-dependent demyristoylation, suggesting that it has pleiotropic nonspecific effects [207]. The quercetin derivative cyanidin has been reported to be an effective activator of SIRT6 that upregulates its deacetylation activity and expression [208]. Ergothioneine (Egt) stimulated SIRT6 enzymatic activity and restricted NF-κB signaling, reducing ROS levels and inhibiting inflammation and senescence [209]. Chrysophanol has been shown to upregulate SIRT6 expression, alleviating metabolic syndromes mediated through the SIRT6/AMPK signaling pathway [210].

Second, many synthesized chemical compounds have been developed to activate SIRT6. For example, UBCS039 increased the deacetylation activity of SIRT6 to enhance autophagy-dependent cell death under oxidative stress [211]. 2-(1-Benzofuran-2-yl)-N-(diphenylmethyl) quinoline-4-carboxamide is another promising activator of SIRT6 that has shown excellent selectivity and biological anticancer functions [212]. Recently, several allosteric SIRT6 deacetylase activators have been identified, such as MDL-800, MDL-801and MDL-811 [213, 214]. Among these compounds, MDL-800 is notable because its permeability coefficient shows that it can be appropriately internalized and accumulated in cells. By activating SIRT6, MDL-800 has been shown to improve genome stability by promoting NHEJ and BER and attenuating hepatocyte injury and fibrosis [29, 215]. MDL-811 activates SIRT6 to attenuate inflammation and ischemic injury via the EZH2-FOXC1 axis [214]. Finally, an increasing number of studies have found that the pansirtuin activator nicotinamide riboside (NR), which upregulates NAD^+^ levels, may be a potential drug for the treatment of SIRT6-related diseases [216].

Third, clinical drugs have been shown to modulate SIRT6 to exert protective effects. A GLP-1 receptor agonist (GLP-1RA) and an SGLT2 inhibitor (SGLT2i) were shown to stabilize plaques in diabetic patients by upregulating SIRT6 [217, 218]. In addition, the hypolipidemic agent fluvastatin regulates cholesterol homeostasis by upregulating the deacetylase activity of SIRT6 [219, 220]. Rosiglitazone and rolipram have been shown to upregulate SIRT6 expression, ameliorating metabolic disorder [221, 222]. Detailed information on SIRT6 activators and their effects is presented in Table 2.

### 5.2 SIRT6 inhibitors

Loss and gain of SIRT6 function result in paradoxical phenotypes in many diseases, such as altered metabolism and inflammation, making it difficult to ascertain whether inhibiting or activating SIRT6 actions is preferable for maintaining homeostasis [15, 73, 111, 160]. Therefore, several SIRT6 inhibitors playing protective roles through their intervention of SIRT6 activity under certain conditions have been developed. Compounds with a quinazoline dione-based structure and OSS-128167 impaired the binding of competing ligands, reducing SIRT6 deacetylase activity [223]. Among these compounds, SYN17739303 lowered blood glucose and inhibited inflammatory responses [114], while OSS-128167 upregulated glucose uptake and inhibited TNF-α activity in a time-dependent manner, sensitizing cancer cells to DNA-damaging agents [223].

**Table 2:** The activators of SIRT6.

Categories and names			Mechanisms	Functions	Ref.
Natural products	Icariin 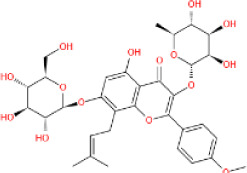		Promotes SIRT6 expression and enzymatic activity; inhibits NF-κB signaling	Attenuate senescence and cardiac inflammation	[198]
	Egt 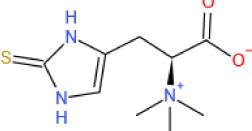		Promotes SIRT6 expression; inhibits NF-κB signaling	Attenuates high glucose-induced endothelial senescence	[209]
	Flavonoids	Quercetin 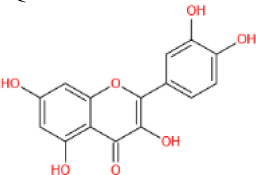	Binds at a distal region of the acyl channel to promote SIRT6 deacetylase activity (EC50=990±250 μM)	Inhibits frizzled 4 (FZD4) expression to repress proliferation and invasion of hepatoblastoma cells	[207, 253]
		Cyanidin 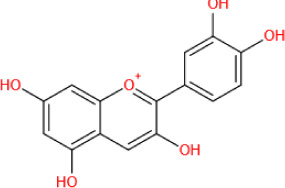	Binds at a distal region of the acyl channel to promote SIRT6 expression and deacetylase activity (EC50=460±20 μM); promotes FoxO3α, reduces GLUT1 and NF-κB signaling	Reduces inflammatory reactions	[208, 254]
	Chrysophanol 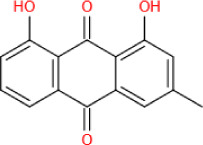		Promotes SIRT6 expression; promotes SIRT6/AMPK signaling	Alleviates metabolic disorders	[210]
Chemical synthesized compounds	UBCS039 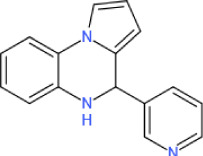		Binds at a distal region of the acyl channel and “C site” of SIRT6 to promote SIRT6 deacetylase activity (EC50=38±13 μM)	Promotes autophagy under oxidative stress	[207, 211, 255]
	2-(1-benzofuran-2-yl)-N-(diphenylmethyl) quinoline-4-carboxamide 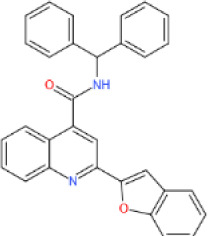		Binds the surface allosteric site of SIRT6 to promote deacetylase (EC50=5.35±0.69 μM) and demyristoylase (EC50= 0.72 ± 0.25 μM) activities	Inhibits tumor growth	[212]
	MDL-800 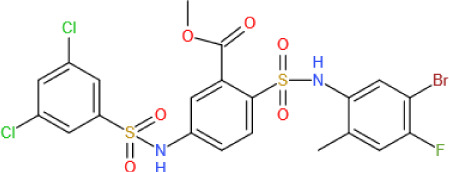		Binds the surface allosteric site of SIRT6 to promote SIRT6 deacetylase activity (EC50=10.3 μM); activates NHEJ and BER	Attenuates hepatocyte injury and fibrosis	[29, 213, 215]
	MDL-811 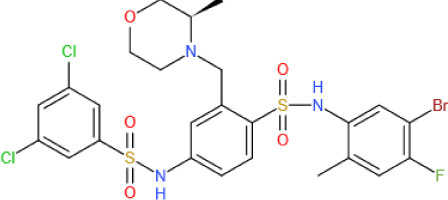		Binds the surface allosteric site of SIRT6 to promote SIRT6 deacetylation activity (EC50=5.7±0.8 μM); promotes EZH2 deacetylation and further FOXC1 expression; inhibits *CYP24A1* expression	Attenuates ischemic injury; inhibits the proliferation of colorectal cancer cells	[214, 256]
	Nicotinamide riboside 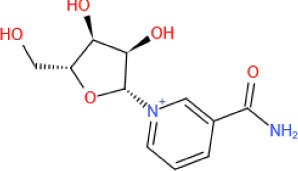		Promotes sirtuin deacetylase activity by increasing NAD^+^ levels	Attenuates SIRT6 (or sirtuin)-related diseases	[216]
Clinical drugs	GLP-1RA and SGLT2i		Promotes SIRT6 expression	Inhibits inflammation and oxidative in ECs and stabilizes atherosclerotic plaque	[217, 218]
	Fluvastatin 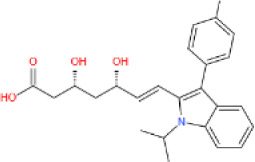		Binds the exit of the SIRT6 acyl channel; increases the phosphorylation of SREBP1 and AMPKα	Maintains cholesterol homeostasis	[219, 220]
	Rosiglitazone 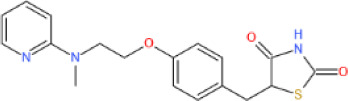		Promotes SIRT6 expression by activating PPARγ	Ameliorates hepatic lipid accumulation	[222]
	Rolipram 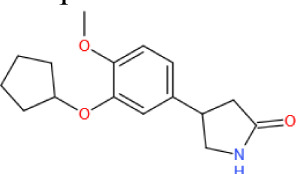		Promotes SIRT6 expression	Improves adipose distribution and metabolic disorder	[221]

Recently, trichostatin A (TSA), a known inhibitor of class I/II HDACs, has been shown to inhibit SIRT6 deacetylase activity with higher specificity and affinity than other known inhibitors [224, 225]. TSA increased p53K382Ac levels, accelerating the p53-mediated apoptosis [224]. Similar to quercetin, the quercetin derivative catechin gallate (CG) occupies the acyl channel of SIRT6, but powerfully inhibits SIRT6 activity [207]. By screening DNA-encoded chemical libraries, scientists found that the compound A127-(CONHPr)-B178 effectively inhibited SIRT6 activity, as shown by increased DNA damage, accelerated apoptosis and decreased TNF-α secretion [226]. Synthesized peptides harboring a different N^ε^-modified lysine residue mimicked endogenous substrates to competitively occupy substrate-binding regions, thereby inhibiting SIRT6 deacetylase and/or demyristoylase activity [227-230]. In a mouse model of T2DM, the compound 5-(4-methylpiperazin-1-yl)-2-nitroaniline upregulated GLUT1 expression to improve blood glucose levels by inhibiting SIRT6 deacetylase activity [115]. SIRT6 inhibitors and their effects are presented in Table 3.

Overall, the development of modulators targeting SIRT6 provides novel and promising strategies for the treatment of human diseases. Despite the limited number of clinical trials on SIRT6 modulators, several modulators, such as MDL-800, DML-811 and quinazoline dione analogs, have been shown to exert therapeutic effects in animal experiments. In addition, animal experiments and clinical studies have shown that moderate exercise and calorie restriction (CR) stimulated SIRT6 expression, improving age-related pathological processes such as inflammation, metabolic disorders, and susceptibility to CVDs [231-235]. Therefore, a healthy lifestyle, such as moderate physical exercise and CR, may be potential tools for preventing cellular senescence and aging by regulating SIRT6. In summary, these available interventions can pave the way for scientists to discover additional potent SIRT6 modulators with excellent drug-like properties.

## 6. Future perspectives

A decade of studies on SIRT6 has revealed its expansive functions and simultaneously shown the limitations of our current understanding of SIRT6. SIRT6 regulates chromatin status during transcription and DNA repair [155, 236], as well as transcriptional silencing during DNA repair[34], suggesting that it balances transcription and repair pathways in unexplored ways. A close relationship has been identified between SIRT1 and SIRT6, such as their synergistic effect on DNA repair and antagonistic effect on cancer [59, 237]. In addition, SIRT3 and SIRT6 have been found to maintain each other’s levels, attenuating the development of DCM [128]. Nevertheless, understanding the crosstalk between SIRT6 and other sirtuins, including possible links and cellular mechanisms in various diseases, remains a challenge in this field. In DNA damage repair and CVDs, SIRT6 is closely related to p53: p53 activates the expression of SIRT6 to protect against DNA damage, whereas SIRT6 attenuates doxorubicin (DOX)-induced cardiotoxicity by inhibiting p53 transcriptional activity [238, 239]. Studies have also shown that SIRT6 inhibits p53 transcriptional activity by deacetylating p53 at K381 and K382 [182, 224]. However, further studies are needed to determine whether SIRT6-induced deacetylation of p53 and crosstalk between SIRT6 and p53 play roles in other CVDs. Notably, both glucose deprivation and I/R significantly reduce SIRT6 chromatin enrichment and nuclear content [71, 155], suggesting that the roles of SIRT6 extend outside the nucleus, which is consistent with the roles played by SIRT6 in TNF-α secretion and cytoplasmic stress granule formation [5, 15]. According to these findings, results from further studies on the roles of SIRT6 in regulating the interaction between intranuclear and cytoplasmic factors will be significant.

**Table 3:** The inhibitors of SIRT6.

Categories and names		Mechanisms	Functions	Ref.
Quinazolinediones	SYN17739303 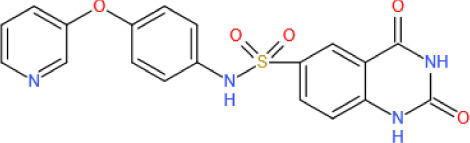	All quinazolinediones bind the NAM-binding pocket, inhibiting the deacetylase activity of SIRT6; increase GLUT1 expression and reduce TNF-α secretion	Improves oral glucose tolerance, lowers blood glucose in T2DM; inhibits inflammatory responses	[114, 223, 257, 258]
Salicylates	OSS-128167 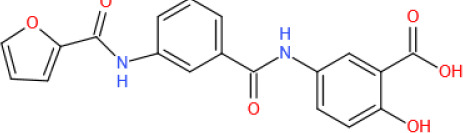	The salicylic moiety interacts with the NAM-binding pocket of SIRT6, inhibiting its deacetylase activity; reduces TNF-α secretion	Increases glucose uptake; sensitizes cancer cells to DNA damage agents; promotes cellular apoptosis in diffuse large B-cell lymphoma cells through inhibiting SIRT6-induced activation of PI3K/Akt/mTOR signaling pathway	[223, 259]
Trichostatin A	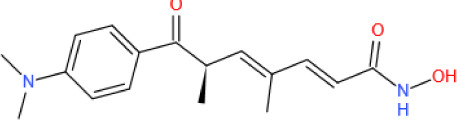	Binds the NAM pocket and acyl channel to inhibit SIRT6 deacetylase activity; increases p53K382 acetylation level	Increasing cellular apoptosis under stress resistance	[224, 225]
Catechin gallate	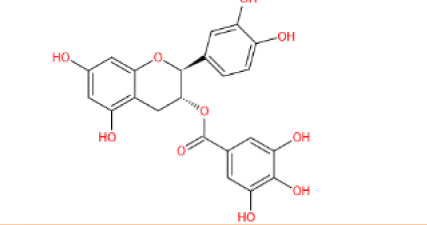	Binds the acyl channel to inhibit SIRT6 deacetylase activity	Not reported	[207]
A127-(CONHPr)-B178	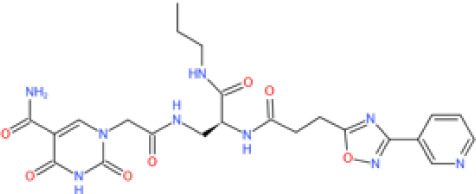	Binds the NAD^+^ pocket to inhibit demyristoylase activity (IC_50_=6.7 μmol)	Promotes DNA damage, senescence and cell apoptosis	[226]
Lysine-based peptides	BH-TM4 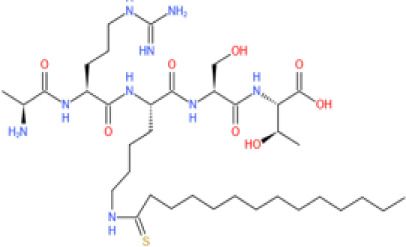	A modified lysine residue binds the N^ε^-acetyl lysine-binding site, inhibiting both the deacetylase (IC_50_=8.2 μmol) and demyristoylase (IC_50_=1.7 μmol) activities of SIRT6	Not reported	[229]
	Cyclic peptide 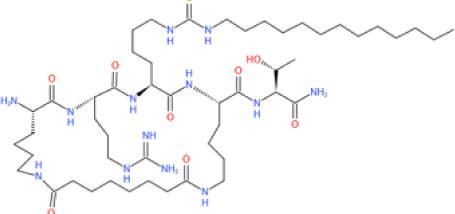	Inhibits the demyristoylase activity (IC_50_=0.319 μmol) of SIRT6	Not reported	[230]
5-(4-Methylpiperazin-1-yl)-2-nitroaniline	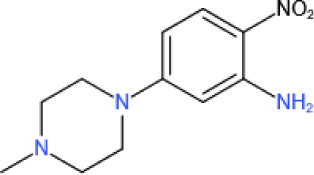	Inhibits SIRT6 deacetylase activity (IC_50_= 4.93 μmol); promotes GLUT1 levels	Reduces blood glucose	[115]

To date, three distinctive enzymatic activities of SIRT6 have been elucidated: deacetylation, mono-ADP-ribosylation and deacylation of long-chain fatty acyl groups, such as myristoyl. The histone deacetylase activity of SIRT6 has been well characterized even though it is weaker *in vitro* than that of other sirtuins, which may be attributed to improved methods of detecting acetylated substrates [9]. Although studies have found that SIRT6 preferentially engages in long chain fatty acylation and that SIRT6 exhibits more powerful fatty acid deacylase activity than deacetylation activity *in vitro*, only a few substrates of SIRT6 fatty deacylation have been detected *in vivo* [15, 23, 38]. This dearth in substrate identification is partially due to the lack of efficient enrichment methods for lysine fatty-acylated peptides, which has limited the detection of these substrates to some extent. Therefore, developing pan antibodies against fatty acyl groups or identifying efficient fatty acyl-binding proteins may promote the development of new enrichment methods to detect substrates of SIRT6 fatty deacylation. In addition, activating the mono-ADP-ribosylation activity of SIRT6 may require specific conditions *in vivo[9]*, as indicated by studies reporting that SIRT6 seems more likely to exhibit this activity against nuclear proteins under oxidative stress [32-35]. This requirement raises the following two questions: is the mono-ADP-ribosylation activity of SIRT6 related to its subcellular localization or stress conditions, and how does SIRT6 balance its three enzymatic activities and the crosstalk among the related substrates under physiological or stress conditions? Finally, recent reports have shown that the nonenzymatic activities of SIRT6 are also important [190, 195, 197]. Therefore, further studies are needed to reveal other significant deacetylase-independent roles played by SIRT6.

Although the significance of SIRT6 in human diseases is known to a certain extent, the number of SIRT6 modulators is still tiny. Indeed, only limited regulatory activity and efficacy in cells and animal models have been successfully observed because these modulators do not have optimal drug properties. Among known activators, pansirtuin activators may not be suitable for human use, as SIRT4 may exert detrimental effects in people with CVDs, such as aggravation of pathological cardiac hypertrophy [240]. Additionally, other phenomena need to be further studied: depletion and supraphysiological expression of SIRT6 in POMC neurons that disrupt both POMC production and POMC-related metabolism [141, 142], and overexpression of SIRT6 that aggravates the instability of atherosclerotic plaques [9, 72]. In addition, activators of SIRT6 have been described as potential tools for treating certain cancers; however, SIRT6 acts as a tumor promoter in some cancer types [241]. Therefore, SIRT6 may engage in cell- and/or tissue-specific regulation of transcription factors to regulate cellular biological processes. The findings have suggested that cell and/or tissue specificity, SIRT6 expression level and disease and disease stage specificity need to be taken into account when SIRT6 modulators are used. Therefore, future studies should explore how drug delivery systems can be utilized to specifically deliver SIRT6 modulators to targeted cells or tissues as needed. Certain SIRT6 inhibitors have been proven effective in lowering blood glucose in a T2DM animal model [114, 223]; however, their effects on other physiological functions within the body, such as aging, inflammation and susceptibility to CVDs, have not been evaluated. More importantly, reduced SIRT6-induced increases in glycolysis leads to tumorigenesis [242] (Supplementary Fig. 1; the detailed information on SIRT6 in cancers and neurodegenerative diseases in the Supplementary Materials). Therefore, the application of SIRT6 inhibitors in the CVD field should be carefully and further investigated. In addition, it is known that inhibition of SIRT6 activity increases the sensitivity of tumor cells to chemotherapeutic agents [243, 244], suggesting that the combination of SIRT6 inhibitors and other drugs is a feasible strategy for treating cancers.

## 7. Conclusions

In summary, the essential roles of SIRT6 in regulating chromatin and nuclear-cytoplasmic signaling pathways important for cellular homeostasis have been well characterized. In terms of genome stability, SIRT6 enhances DNA repair and maintains telomere integrity by regulating DNA repair and chromatin-associated factors, such as PARP1, DDB2, SNF2H and WRN. With respect to cellular metabolism, SIRT6 regulates multiple metabolic processes, including glycolysis, gluconeogenesis, insulin secretion, lipid synthesis, lipolysis and thermogenesis, mainly by regulating the multiple transcriptional activities of HIF1α, FOXO proteins and the PPAR family of transcription factors. In addition, SIRT6 maintains an appropriate inflammatory response by regulating the TNF-α and NF-κB signaling pathways. These functions can influence cellular senescence and aging-related diseases, including CVDs, cancer, and neurodegenerative diseases. Therefore, studying the biological functions of SIRT6 in different diseases is valuable and helpful for the identification of highly specific SIRT6 cellular targets. With a deeper understanding of SIRT6, certain SIRT6 regulatory compounds have been identified, offering novel and promising therapeutic options for aging-related diseases. Notably, recent studies have suggested that SIRT6 exerts adverse effects in some diseases, which are intriguing findings and worthy of further study. Therefore, when SIRT6-targeted therapeutic strategies or modulators are designed, the multifaceted features and cell specificity of SIRT6 need to be considered.

## Supplementary Materials

The Supplementary data can be found online at: www.aginganddisease.org/EN/10.14336/AD.2022.0413.
