# Supplementary material for: SIRT6 in Aging, Metabolism, Inflammation and Cardiovascular Diseases
Source: Aging Dis. 2022 Dec 1;13(6):1787–822. doi: 10.14336/AD.2022.0413 (PMC9662279; doi:10.14336/AD.2022.0413)
Supplement: Supplementary file 1 — The Supplementary data can be found online at: www.aginganddisease.org/EN/10.14336/AD.2022.0413. [file AD-13-6-1787-s.pdf]

SUPPLEMENTARY DATA

SIRT6 in Aging, Metabolism, Inflammation and Cardiovascular Diseases

Zhenyang Guo[1#], Peng Li[1#], Junbo Ge[1,2*], Hua Li[1,2*]

© 2022. Guo Z et al. Published online at http://www.aginganddisease.org/EN/10.14336/AD.2022.0413

SUPPLEMENTARY DATA

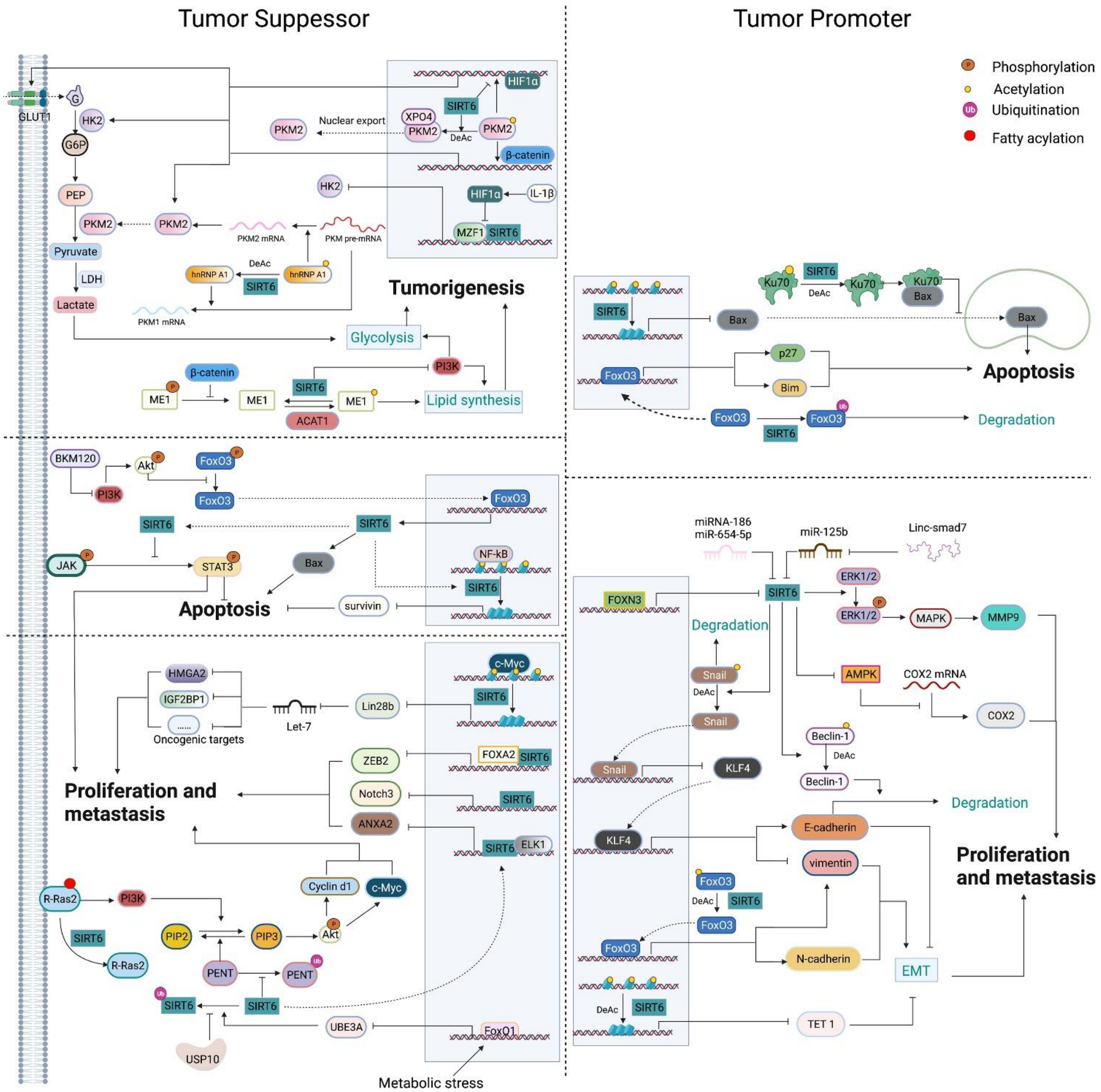

Supplementary Figure 1. SIRT6 regulates the onset and development of cancers SIRT6 inhibits expression of downstream target genes of HIF1α, such as PDK1, LDH, HK2 and PKM2, thereby suppressing glycolysis process. SIRT6 deacetylates PKM2 at K433 to ensure its interaction with XPO4, thereby promoting PKM2 nuclear export and abolishing its functions as transcriptional coactivator of β-catenin. SIRT6 also deacetylates hnRNP A1 at four sites (K3, K52, K87, and K350) to block alternative splicing of PKM mRNA to PKM2, thereby inhibiting PKM2 expression and PKM2-β-catenin signaling pathway. The SIRT6-MZF1 complex on HK2 promoter negatively regulates HK2, while IL-1β-induced HIF-1α abolishes the interaction between SIRT6 and MZF1. SIRT6 deacetylates ME1 at K337 to promote its activity and subsequent lipid synthesis, eventually inhibiting CRC tumorigenesis.

BKM120 inhibits PI3K/Akt signaling to decrease the phosphorylation of FoxO3a, promoting its accumulation on SIRT6 promoter and triggering SIRT6 transcription. On the promoter of survivn, SIRT6 deacetylates H3K9 and reduces NF-κB accumulation to inhibit survivn expression, thereby promoting cancer cells apoptosis. SIRT6 also activates Bax and suppresses JAK/STAT3 signaling pathway to enhance cellular apoptosis. In some cancer cells, however, SIRT6 deacetylates H3K9 within Bax promoter to inhibit Bax expression. SIRT6 also deacetylates Ku70 at K542 to facilitate association between Ku70 and Bax, which blocks Bax translocation to mitochondria

© 2022. Guo Z et al. Published online at http://www.aginganddisease.org/EN/10.14336/AD.2022.0413

and thereby inhibiting cancer cells apoptosis. SIRT6 ubiquitinates FoxO3 to reduce FoxO3 translocation into the nucleus, thereby reducing expression of FoxO3 target genes p27 and Bim and inhibiting cellular apoptosis.

UBE3A ubiquitylates SIRT6 at K160 to promote SIRT6 degradation. Under metabolic stress, FOXO1 transcriptionally represses UBE3A to stabilize SIRT6. Through deacetylating H3K9, SIRT6 acts as a corepressor of ELK1 to suppress ANXA2 expression, thereby reducing the proliferative capacity of cancer cells. SIRT6 inhibits Notch3 signaling pathway to inhibit cellular proliferation and invasion. Coordination of FOXA2 and SIRT6 on the promoter of ZEB2 inhibits its transcription, which resulting in decreased cancer cell progression. SIRT6 also prevents PENT ubiquitylation and degradation, decreasing phosphorylation of PIP3 to form PIP2, inhibiting PI3K/Akt pathway and downstream genes expression such as c-myc and cyclin d1, and eventually repressing cellular proliferation. USP10 stabilizes SIRT6 to antagonize the transcriptional activity of c-myc. SIRT6 also inhibits plasma membrane localization of R-Ras2 through reducing its lysine fatty acylation at four sites (K192, K194, K196, and K197), thereby inactivating PI3K/Akt signaling pathway. SIRT6 represses c-myc-driven transcription of lin28b through deacetylating H3K9 and H3K56, thereby abolishing the suppression of lin28b on Let-7. Let-7 promotes the degradation of oncogenic proteins, such as insulin growth factor 2 binding proteins (IGF2BPs) and high mobility group AT-hook 2 (HMGA2), inhibiting cellular growth.

SIRT6 could upregulate phosphorylation of ERK1/2 to activate MAPK-MMP9 pathway, enhancing cancer invasiveness. FOXN3 downregulates SIRT6 transcription. MiR-186 and miR-654-5p both impair SIRT6 expression to reduce proliferation capacity of cancer cells. On the contrary, Linc-smad7 sponges miR-125b to upregulate SIRT6 expression, promoting cellular proliferation and invasion. SIRT6 downregulates AMPK activity to stabilize mRNA of COX-2, thereby enhancing COX-2 translation and cellular proliferation. SIRT6 deacetylates Snail to prevent its degradation, and then Klf4 expression is suppressed. Klf4 maintains E-cadherin expression and reduces vimentin expression, thereby inhibiting EMT. SIRT6-mediated deacetylation of Beclin-1 promotes autophagic degradation of E-cadherin. SIRT6 deacetylates FoxO3a to promote N-cadherin and Vimentin expression, thereby enhancing EMT. Conversely, through deacetylating H3K9, SIRT6 suppresses TET 1 transcription to accelerate EMT.

1. SIRT6 regulates the onset and development of cancers

In many human cancers, downregulation of SIRT6 expression was reported, which in a large part was associated with increased tumor progression and poor clinical outcome. However, other studies also found that SIRT6 could exert oncogenic functions in a subset of human cancers[1]. Therefore, SIRT6 was considered as a double-edged sword that possesses dual roles of tumor suppressor and promoter (Supplementary Fig. 1).

1.1 Tumorigenesis

Aerobic glycolysis known as the Warburg effect is an important source for energy and macromolecular synthesis in the development and progression of cancer cells. As mentioned above, SIRT6 acts as a corepressor of HIF1α to inhibit glycolysis[2]. In SIRT6-deficient cells, expression levels of glycolytic genes such as PDK1, LDH, hexokinase 2(HK2) and PKM2, were enhanced, and increased aerobic glycolysis promotes oncogenic transformation in an oncogene activation-independent manner[3]. On the upstream, FoxO3a and RUNX2 could upregulate and downregulate SIRT6 transcription, respectively, to regulate glycolysis and subsequent cell differentiation, viability, and clinical outcome[4,5]. As a key rate-limiting enzyme of aerobic glycolysis, the overexpression of PKM2 contributes to tumorigenesis[6]. SIRT6 was shown to deacetylate heterogeneous nuclear ribonucleoproteins A1(hnRNP A1) to block alternative splicing of PKM mRNA to PKM2, reducing PKM2 expression[7]. In addition, deacetylation of PKM2 by SIRT6 led to the nuclear export of PKM2, thereby abrogating its non-glycolytic functions (i.e., nuclear protein kinase and transcriptional coactivator functions)[8]. SIRT6 was also shown to be a suppressor of HK2, regulating inflammation and glycolysis in the tumor microenvironment[9]. Inhibition of PI3K signaling by SIRT6 at the transcriptional level suppresses glycolysis and lipid metabolism in cancer stem cells, antagonizing tumor sphere formation[10]. In addition, SIRT6 antagonizes acetyl-CoA acetyltransferase (ACAT1) function to inhibit malic enzyme 1 (ME1) activity, which reduces NADPH for fatty acid biosynthesis, eventually suppressing colorectal tumorigenesis[11]. However, in papillary thyroid cancer cells, SIRT6 was shown to upregulate glycolytic genes expression and Warburg effect via upregulation of ROS[12].

1.2 Proliferation and metastasis of cancer cells

Uncontrolled cellular proliferation is one of the hallmarks of cancer progression. Upon metabolic stress, FoxO1 transcriptionally represses the expression of E3 ubiquitin ligase UBE3A to inhibit degradation of SIRT6 by UBE3A, which, in turn, results in *Annexin A2* (*ANXA2)* repression, eventually reducing the proliferative capacity and invasiveness of HCC[13]. In colon cancer, USP10 stabilizes SIRT6 to antagonize the transcriptional activity of the c-myc,

and then induces cell cycle arrest and tumor growth inhibition[14]. In addition, SIRT6 increases expression and stability of PENT to disrupt PI3K/AKT signaling, suppressing proliferation and invasion of colon cancer cells[15]. SIRT6 also decreases the lysine fatty acylation of R-Ras2 to reduce its membrane localization and subsequent activation of PI3K/AKT signaling, suppressing cellular proliferation[16]. In addition, SIRT6 downregulation is associated with poor prognosis of pancreatic ductal adenocarcinoma (PDAC)[17]. Loss of SIRT6 results in hyperacetylation of histone and c-myc recruitment within the Lin28b promoter to upregulate expression of Lin28b and downstream let-7 target genes, promoting proliferation and progression of PDAC[17]. Downregulation of SIRT6 by miR-34c-5p enhances cell proliferation through activating JAK2/STAT3 signaling pathway[18]. In addition, SIRT6 represses proliferation and invasion through inhibiting Notch3 signaling pathway[19,20]. SIRT6 also positively regulates the levels of the phosphorylated extracellular signal-regulated kinases 1 and 2 (pERK1/2) to activate matrix metalloproteinase-9 (MMP-9) signaling pathway, promoting cell growth and metastasis in bone cancer[21]. However, forkhead box N3 (FOXN3) transcriptionally inhibits the SIRT6 expression, thereby repressing MMP9 to downregulate proliferation in osteosarcoma[22]. In human squamous cell carcinoma, UVB upregulates SIRT6 expression via activating the AKT pathway, and then SIRT6 promotes the expression of COX-2, increasing cell survival and proliferation[23]. Downregulated SIRT6 by miRNA-186 and miR-654-5p contribute to impaired proliferation capacity of cancer cells[24,25].

1.3 Epithelial–mesenchymal transition of cancer cells

Epithelial–mesenchymal transition (EMT) confers malignant properties, such as invasiveness and metastasis, to carcinoma cells[26]. SIRT6-mediated invasiveness is significantly related to the expression of EMT associated molecules, such as E-cadherin, N-cadherin, vimentin, Snail and activated β-catenin[27,28]. Mechanistically, SIRT6 not only deacetylates Beclin-1 to enhance the autophagic degradation of E-cadherin, but also deacetylates FoxO3 to promote N-cadherin and vimentin expression, eventually promoting EMT of HCC[29]. In addition, long intergenic noncoding RNA smad7 (Linc-smad7) upregulates SIRT6 through sponging miR-125b, promoting EMT of HCC[30]. SIRT6 also promotes the deacetylation and stabilization of Snail to repress the expression of *Klf4*[31]. Subsequently, reduced KLF4 results in decreased E-cadherin expression and increased vimentin expression, promoting EMT and aggressiveness of non-small cell lung cancer[31]. In colon carcinoma cells, SIRT6 suppresses tet methylcytosine dioxygenase 1 (TET 1) transcription through reducing H3K9 deacetylation to accelerate EMT[32]. However, in PDAC cells, transcriptionally upregulated expression of SIRT6 by KLF10 was shown to ameliorate glycolysis, EMT and metastasis[33]. In addition, coordination of SIRT6 and FOXA2 inhibits the expression of zinc finger E-box binding homeobox 2 (ZEB2) to suppress proliferation and invasion of HCC[34].

1.4 Apoptosis of cancer cells

Cellular apoptosis is an important part of tumor development, therefore, therapies triggering apoptosis have been used to eliminate malignant cells[35]. Early animal studies found that SIRT6 could inhibit the liver cancer development at the initiation stage through reducing the antiapoptotic activity of survivn via reducing both H3K9Ac and NF-κB levels at the survivin promoter[36]. Furthermore, the mono-ADP ribosyltransferase activity of SIRT6 also potentiates apoptosis in cancer cells line through ATM-mediated p53 and p73 signalling cascades, but not in normal cells[37]. Interestingly, unlike SIRT6 in proliferation, SIRT6 was shown to block the ERK signaling pathway to induce cancer cell apoptosis[38]. In glioma cells, SIRT6 induces apoptosis through activating the JAK2/STAT3 signaling pathway[39]. In colorectal cancer cells, PI3K inhibitor BKM120 was found to positively regulate SIRT6 expression by reducing phosphorylation level of FoxO3a, which in turn promotes apoptosis through activating Bax and mitochondrial pathway[40]. However, SIRT6 could also play oncogenic roles through attenuating Bax signaling to potentiate apoptosis evasion in HCC[41,42]. Mechanistically, on one hand, SIRT6 could inhibit Bax expression through deacetylating H3K9; on the other hand, SIRT6 deacetylates Ku70 to promote Bax-Ku70 interaction, thereby blocking Bax mitochondrial translocation[41,42]. In addition, SIRT6 prevents FoxO3 translocation into the nucleus, which reduces expression of its target genes p27 and Bim, thereby preventing doxorubicin-induced cell death[43].

Collectively, SIRT6 fulfills a controversial role in regulating complex network of biological pathways in different cancer cells, which is partly due to the high heterogeneity of tumor cells. In general, SIRT6 protects against tumorigenesis, while roles of SIRT6 in cancer are difficult to identified after tumor formation. Though this pleiotropism of SIRT6 adds a layer of difficulty in understanding the cellular mechanisms by which SIRT6 impacts biological

© 2022. Guo Z et al. Published online at http://www.aginganddisease.org/EN/10.14336/AD.2022.0413

processes of cancer cells, opportunities appear to be encouraging for attenuating cancer development through targeting SIRT6. Several studies have shown that the sensitivity of tumor cells to chemotherapy or immunotherapy can be improved through inhibiting SIRT6[44-47]. Therefore, targeting SIRT6 seems a novel and promising strategy to improve cancer treatment and patient outcome.

2. Neurodegenerative diseases

Alzheimer's disease (AD) and Parkinson's disease (PD) are common ageing-related diseases. AD is involved in neurodegeneration, and characterized by the deposition of Amyloid β (Aβ) and the accumulation of hyperphosphorylated Tau at the cellular level[48]. SIRT6 reduction was significantly observed in both AD mouse model and patients, implying that the aberration of SIRT6 is closely associated with pathologies of AD[49,50]. In detail, SIRT6 could prevent Aβ42-induced DNA damage, attenuating development of AD[50]. In addition, SIRT6 deficiency results in glycogen synthase kinase 3 (GSK3) activation to increase stability and phosphorylation of Tau protein, a critical mark in neurodegenerative diseases[49]. SIRT6 also deacetylates Tau at K174 to decrease its stability and nuclear accumulation, attenuating DNA damage and nucleolar dysfunction[51]. A recent study shows that bolstering neuronal NAD^+ levels could increase SIRT6 activity, which improves synaptic dysfunction, and neuronal degeneration through attenuating DNA damage, phosphorylation of Tau (pTau) accumulation and neuroinflammation[52]. However, SIRT6 level was reported to be greater in PD patient brains, and in which, SIRT6 plays pathogenic roles through upregulating the pro-apoptotic TNF-α pathway and downregulating the pro-survival AKT signalling[53]. In addition, a recent study shows that mRNA level of SIRT6 was up-regulated in the peripheral blood of PD patients[54], suggesting that SIRT6 may play a pathogenic role in PD development.

In sum, current studies on SIRT6 in neurodegenerative diseases are limited, and SIRT6 seems to play contradictory roles in AD and PD. This discrepancy may be contributed to cell specificity. But in any case, SIRT6 is undoubtedly involved in neurodegenerative diseases, therefore, more research for understanding its specific mechanisms is needed.

References

1 Tasselli, L., Zheng, W. & Chua, K. F. SIRT6: Novel Mechanisms and Links to Aging and Disease. *Trends Endocrinol Metab* **28**, 168-185, doi:10.1016/j.tem.2016.10.002 (2017).

2 Zhong, L. *et al.* The histone deacetylase Sirt6 regulates glucose homeostasis via Hif1alpha. *Cell* **140**, 280-293, doi:10.1016/j.cell.2009.12.041 (2010).

3 Sebastián, C. *et al.* The histone deacetylase SIRT6 is a tumor suppressor that controls cancer metabolism. *Cell* **151**, 1185-1199, doi:10.1016/j.cell.2012.10.047 (2012).

4 Dong, Z. *et al.* FOXO3a-SIRT6 axis suppresses aerobic glycolysis in melanoma. *Int J Oncol* **56**, 728-742, doi:10.3892/ijo.2020.4964 (2020).

5 Choe, M. *et al.* The RUNX2 Transcription Factor Negatively Regulates SIRT6 Expression to Alter Glucose Metabolism in Breast Cancer Cells. *J Cell Biochem* **116**, 2210-2226, doi:10.1002/jcb.25171 (2015).

6 Luo, W. *et al.* Pyruvate kinase M2 is a PHD3-stimulated coactivator for hypoxia-inducible factor 1. *Cell* **145**, 732-744, doi:10.1016/j.cell.2011.03.054 (2011).

7 Yang, H. *et al.* Sirtuin-mediated deacetylation of hnRNP A1 suppresses glycolysis and growth in hepatocellular carcinoma. *Oncogene* **38**, 4915-4931, doi:10.1038/s41388-019-0764-z (2019).

8 Bhardwaj, A. & Das, S. SIRT6 deacetylates PKM2 to suppress its nuclear localization and oncogenic functions. *Proc Natl Acad Sci U S A* **113**, E538-547, doi:10.1073/pnas.1520045113 (2016).

9 Gupta, P., Sheikh, T. & Sen, E. SIRT6 regulated nucleosomal occupancy affects Hexokinase 2 expression. *Exp Cell Res* **357**, 98-106, doi:10.1016/j.yexcr.2017.05.005 (2017).

10 Ioris, R. M. *et al.* SIRT6 Suppresses Cancer Stem-like Capacity in Tumors with PI3K Activation Independently of Its Deacetylase Activity. *Cell Rep* **18**, 1858-1868, doi:10.1016/j.celrep.2017.01.065 (2017).

11 Zhu, Y. *et al.* Dynamic Regulation of ME1 Phosphorylation and Acetylation Affects Lipid Metabolism and Colorectal Tumorigenesis. *Mol Cell* **77**, 138-149.e135, doi:10.1016/j.molcel.2019.10.015 (2020).

12 Yu, W., Yang, Z., Huang, R., Min, Z. & Ye, M. SIRT6 promotes the Warburg effect of papillary thyroid cancer cell BCPAP through reactive oxygen species. *Onco Targets Ther* **12**, 2861-2868, doi:10.2147/ott.S194256 (2019).

13 Kohli, S., Bhardwaj, A., Kumari, R. & Das, S. SIRT6 Is a Target of Regulation by UBE3A That Contributes to Liver Tumorigenesis in an ANXA2-Dependent Manner. *Cancer Res* **78**, 645-658, doi:10.1158/0008-5472.Can-17-1692 (2018).
14 Lin, Z. *et al.* USP10 antagonizes c-Myc transcriptional activation through SIRT6 stabilization to suppress tumor formation. *Cell Rep* **5**, 1639-1649, doi:10.1016/j.celrep.2013.11.029 (2013).
15 Tian, J. & Yuan, L. Sirtuin 6 inhibits colon cancer progression by modulating PTEN/AKT signaling. *Biomed Pharmacother* **106**, 109-116, doi:10.1016/j.biopha.2018.06.070 (2018).
16 Zhang, X., Spiegelman, N. A., Nelson, O. D., Jing, H. & Lin, H. SIRT6 regulates Ras-related protein R-Ras2 by lysine defatty-acylation. *Elife* **6**, doi:10.7554/eLife.25158 (2017).
17 Kugel, S. *et al.* SIRT6 Suppresses Pancreatic Cancer through Control of Lin28b. *Cell* **165**, 1401-1415, doi:10.1016/j.cell.2016.04.033 (2016).
18 Li, N. *et al.* Downregulation of SIRT6 by miR-34c-5p is associated with poor prognosis and promotes colon cancer proliferation through inhibiting apoptosis via the JAK2/STAT3 signaling pathway. *Int J Oncol* **52**, 1515-1527, doi:10.3892/ijo.2018.4304 (2018).
19 Chen, X., Li, D., Gao, Y., Cao, Y. & Hao, B. Histone deacetylase SIRT6 inhibits glioma cell growth through down-regulating NOTCH3 expression. *Acta Biochim Biophys Sin (Shanghai)* **50**, 417-424, doi:10.1093/abbs/gmy019 (2018).
20 Zhang, J. *et al.* The histone deacetylase SIRT6 inhibits ovarian cancer cell proliferation via down-regulation of Notch 3 expression. *Eur Rev Med Pharmacol Sci* **19**, 818-824 (2015).
21 Lin, H., Hao, Y., Zhao, Z. & Tong, Y. Sirtuin 6 contributes to migration and invasion of osteosarcoma cells via the ERK1/2/MMP9 pathway. *FEBS Open Bio* **7**, 1291-1301, doi:10.1002/2211-5463.12265 (2017).
22 Xue, W., Ma, L., Wang, Z., Zhang, W. & Zhang, X. FOXN3 is downregulated in osteosarcoma and transcriptionally regulates SIRT6, and suppresses migration and invasion in osteosarcoma. *Oncol Rep* **41**, 1404-1414, doi:10.3892/or.2018.6878 (2019).
23 Ming, M. *et al.* SIRT6 promotes COX-2 expression and acts as an oncogene in skin cancer. *Cancer Res* **74**, 5925-5933, doi:10.1158/0008-5472.Can-14-1308 (2014).
24 Ruan, L., Chen, J., Ruan, L., Yang, T. & Wang, P. MicroRNA-186 suppresses lung cancer progression by targeting SIRT6. *Cancer Biomark* **21**, 415-423, doi:10.3233/cbm-170650 (2018).
25 Xu, X. Z., Song, H., Zhao, Y. & Zhang, L. MiR-654-5p regulated cell progression and tumor growth through targeting SIRT6 in osteosarcoma. *Eur Rev Med Pharmacol Sci* **24**, 3517-3525, doi:10.26355/eurrev_202004_20811 (2020).
26 Lu, W. & Kang, Y. Epithelial-Mesenchymal Plasticity in Cancer Progression and Metastasis. *Dev Cell* **49**, 361-374, doi:10.1016/j.devcel.2019.04.010 (2019).
27 Bae, J. S. *et al.* SIRT6 Is Involved in the Progression of Ovarian Carcinomas via β-Catenin-Mediated Epithelial to Mesenchymal Transition. *Front Oncol* **8**, 538, doi:10.3389/fonc.2018.00538 (2018).
28 Yang, Z., Yu, W., Huang, R., Ye, M. & Min, Z. SIRT6/HIF-1α axis promotes papillary thyroid cancer progression by inducing epithelial-mesenchymal transition. *Cancer Cell Int* **19**, 17, doi:10.1186/s12935-019-0730-4 (2019).
29 Han, L. L., Jia, L., Wu, F. & Huang, C. Sirtuin6 (SIRT6) Promotes the EMT of Hepatocellular Carcinoma by Stimulating Autophagic Degradation of E-Cadherin. *Mol Cancer Res* **17**, 2267-2280, doi:10.1158/1541-7786.Mcr-19-0321 (2019).
30 Han, L., Jia, L. & Zan, Y. Long intergenic noncoding RNA smad7 (Linc-smad7) promotes the epithelial-mesenchymal transition of HCC by targeting the miR-125b/SIRT6 axis. *Cancer Med* **9**, 9123-9137, doi:10.1002/cam4.3515 (2020).
31 Li, Z. *et al.* SIRT6 drives epithelial-to-mesenchymal transition and metastasis in non-small cell lung cancer via snail-dependent transrepression of KLF4. *J Exp Clin Cancer Res* **37**, 323, doi:10.1186/s13046-018-0984-z (2018).
32 Geng, C. H. *et al.* Overexpression of Sirt6 is a novel biomarker of malignant human colon carcinoma. *J Cell Biochem* **119**, 3957-3967, doi:10.1002/jcb.26539 (2018).
33 Tsai, Y. C. *et al.* Upregulating sirtuin 6 ameliorates glycolysis, EMT and distant metastasis of pancreatic adenocarcinoma with krüppel-like factor 10 deficiency. *Exp Mol Med* **53**, 1623-1635, doi:10.1038/s12276-021-00687-8 (2021).
34 Liu, J. *et al.* Coordination of FOXA2 and SIRT6 suppresses the hepatocellular carcinoma progression through

© 2022. Guo Z et al. Published online at http://www.aginganddisease.org/EN/10.14336/AD.2022.0413

ZEB2 inhibition. *Cancer Manag Res* **10**, 391-402, doi:10.2147/cmar.S150552 (2018).
35 Mohammad, R. M. *et al.* Broad targeting of resistance to apoptosis in cancer. *Semin Cancer Biol* **35 Suppl**, S78-s103, doi:10.1016/j.semcancer.2015.03.001 (2015).
36 Min, L. *et al.* Liver cancer initiation is controlled by AP-1 through SIRT6-dependent inhibition of survivin. *Nat Cell Biol* **14**, 1203-1211, doi:10.1038/ncb2590 (2012).
37 Van Meter, M., Mao, Z., Gorbunova, V. & Seluanov, A. SIRT6 overexpression induces massive apoptosis in cancer cells but not in normal cells. *Cell Cycle* **10**, 3153-3158, doi:10.4161/cc.10.18.17435 (2011).
38 Zhang, Z. G. & Qin, C. Y. Sirt6 suppresses hepatocellular carcinoma cell growth via inhibiting the extracellular signal-regulated kinase signaling pathway. *Mol Med Rep* **9**, 882-888, doi:10.3892/mmr.2013.1879 (2014).
39 Feng, J. *et al.* SIRT6 suppresses glioma cell growth via induction of apoptosis, inhibition of oxidative stress and suppression of JAK2/STAT3 signaling pathway activation. *Oncol Rep* **35**, 1395-1402, doi:10.3892/or.2015.4477 (2016).
40 Zhang, Y. *et al.* SIRT6, a novel direct transcriptional target of FoxO3a, mediates colon cancer therapy. *Theranostics* **9**, 2380-2394, doi:10.7150/thno.29724 (2019).
41 Tao, N. N. *et al.* Deacetylation of Ku70 by SIRT6 attenuates Bax-mediated apoptosis in hepatocellular carcinoma. *Biochem Biophys Res Commun* **485**, 713-719, doi:10.1016/j.bbrc.2017.02.111 (2017).
42 Ran, L. K. *et al.* SIRT6 Overexpression Potentiates Apoptosis Evasion in Hepatocellular Carcinoma via BCL2-Associated X Protein-Dependent Apoptotic Pathway. *Clin Cancer Res* **22**, 3372-3382, doi:10.1158/1078-0432.Ccr-15-1638 (2016).
43 Hu, J. Q. *et al.* Histone deacetylase SIRT6 regulates chemosensitivity in liver cancer cells via modulation of FOXO3 activity. *Oncol Rep* **40**, 3635-3644, doi:10.3892/or.2018.6770 (2018).
44 Song, L. *et al.* Icariin-induced inhibition of SIRT6/NF-κB triggers redox mediated apoptosis and enhances anti-tumor immunity in triple-negative breast cancer. *Cancer Sci* **111**, 4242-4256, doi:10.1111/cas.14648 (2020).
45 Cea, M. *et al.* Evidence for a role of the histone deacetylase SIRT6 in DNA damage response of multiple myeloma cells. *Blood* **127**, 1138-1150, doi:10.1182/blood-2015-06-649970 (2016).
46 Cagnetta, A. *et al.* Depletion of SIRT6 enzymatic activity increases acute myeloid leukemia cells' vulnerability to DNA-damaging agents. *Haematologica* **103**, 80-90, doi:10.3324/haematol.2017.176248 (2018).
47 Strub, T. *et al.* SIRT6 haploinsufficiency induces BRAF(V600E) melanoma cell resistance to MAPK inhibitors via IGF signalling. *Nat Commun* **9**, 3440, doi:10.1038/s41467-018-05966-z (2018).
48 Bakota, L. & Brandt, R. Tau Biology and Tau-Directed Therapies for Alzheimer's Disease. *Drugs* **76**, 301-313, doi:10.1007/s40265-015-0529-0 (2016).
49 Kaluski, S. *et al.* Neuroprotective Functions for the Histone Deacetylase SIRT6. *Cell Rep* **18**, 3052-3062, doi:10.1016/j.celrep.2017.03.008 (2017).
50 Jung, E. S. *et al.* p53-dependent SIRT6 expression protects Aβ42-induced DNA damage. *Sci Rep* **6**, 25628, doi:10.1038/srep25628 (2016).
51 Portillo, M. *et al.* SIRT6-CBP-dependent nuclear Tau accumulation and its role in protein synthesis. *Cell Rep* **35**, 109035, doi:10.1016/j.celrep.2021.109035 (2021).
52 Hou, Y. *et al.* NAD(+) supplementation normalizes key Alzheimer's features and DNA damage responses in a new AD mouse model with introduced DNA repair deficiency. *Proc Natl Acad Sci U S A* **115**, E1876-e1885, doi:10.1073/pnas.1718819115 (2018).
53 Nicholatos, J. W. *et al.* Nicotine promotes neuron survival and partially protects from Parkinson's disease by suppressing SIRT6. *Acta Neuropathol Commun* **6**, 120, doi:10.1186/s40478-018-0625-y (2018).
54 Maszlag-Török, R., Boros, F. A., Vécsei, L. & Klivényi, P. Gene variants and expression changes of SIRT1 and SIRT6 in peripheral blood are associated with Parkinson's disease. *Sci Rep* **11**, 10677, doi:10.1038/s41598-021-90059-z (2021).

© 2022. Guo Z et al. Published online at http://www.aginganddisease.org/EN/10.14336/AD.2022.0413
